# LY2087101 and dFBr share transmembrane binding sites in the (α4)3(β2)2 Nicotinic Acetylcholine Receptor

**DOI:** 10.1038/s41598-018-19790-4

**Published:** 2018-01-19

**Authors:** Farah Deba, Hamed I. Ali, Abisola Tairu, Kara Ramos, Jihad Ali, Ayman K. Hamouda

**Affiliations:** Department of Pharmaceutical Sciences, Rangel College of Pharmacy, Texas A&M Health Sciences Center, Kingsville, TX 78363 USA

## Abstract

Positive allosteric modulators (PAMs) of nicotinic acetylcholine receptors (nAChRs) have potential therapeutic application in neuropathologies associated with decrease in function or loss of nAChRs. In this study, we characterize the pharmacological interactions of the nAChRs PAM, LY2087101, with the α4β2 nAChR using mutational and computational analyses. LY2087101 potentiated ACh-induced currents of low-sensitivity (α4)3(β2)2 and high-sensitivity (α4)2(β2)3 nAChRs with similar potencies albeit to a different maximum potentiation (potentiation *I*_*max*_ = ~840 and 450%, respectively). Amino acid substitutions within the α4 subunit transmembrane domain [e.g. α4Leu256 and α4Leu260 within the transmembrane helix 1 (TM1); α4Phe316 within the TM3; and α4Gly613 within TM4] significantly reduced LY2087101 potentiation of (α4)3(β2)2 nAChR. The locations of these amino acid residues and LY2087101 computational docking analyses identify two LY2087101 binding sites: an intrasubunit binding site within the transmembrane helix bundle of α4 subunit at the level of α4Leu260/α4Phe316 and intersubunit binding site at the α4:α4 subunit interface at the level of α4Leu256/α4Ile315 with both sites extending toward the extracellular end of the transmembrane domain. We also show that desformylflustrabromine (dFBr) binds to these two sites identified for LY2087101. These results provide structural information that are pertinent to structure-based design of nAChR allosteric modulators.

## Introduction

Nicotinic acetylcholine receptors (nAChRs) are membrane proteins from the Cys-loop family of pentameric ligand-gated ion channels (pLGICs) superfamily which also includes the serotonin 5-hydroxytryptamine type 3 receptor (5-HT3R), the γ-aminobutyric acid type A receptor (GABAAR), and the glycine receptor (GlyR). Neuronal nAChRs are expressed at presynaptic and postsynaptic membranes of cholinergic and other neurotransmitter synapses throughout the nervous system as well as in non-neuronal tissues^1^. They mediate ACh signaling through gating a transmembrane cationic channel which results in fast synaptic transmission and/or regulation of neurotransmitter release^2,3^. Neuronal nAChRs contribute to important brain functions including attention, learning and memory and mediate the rewarding and aversive effects of nicotine, the major addictive component in tobacco products^4^. Abnormalities and/or decrease in the number of neuronal nAChRs have been linked to pathophysiological conditions including cognitive deficits associated with neuropsychiatric disease such as Alzheimer’s and Parkinson’s diseases, schizophrenia and epilepsy^5^.

**Figure 1:**
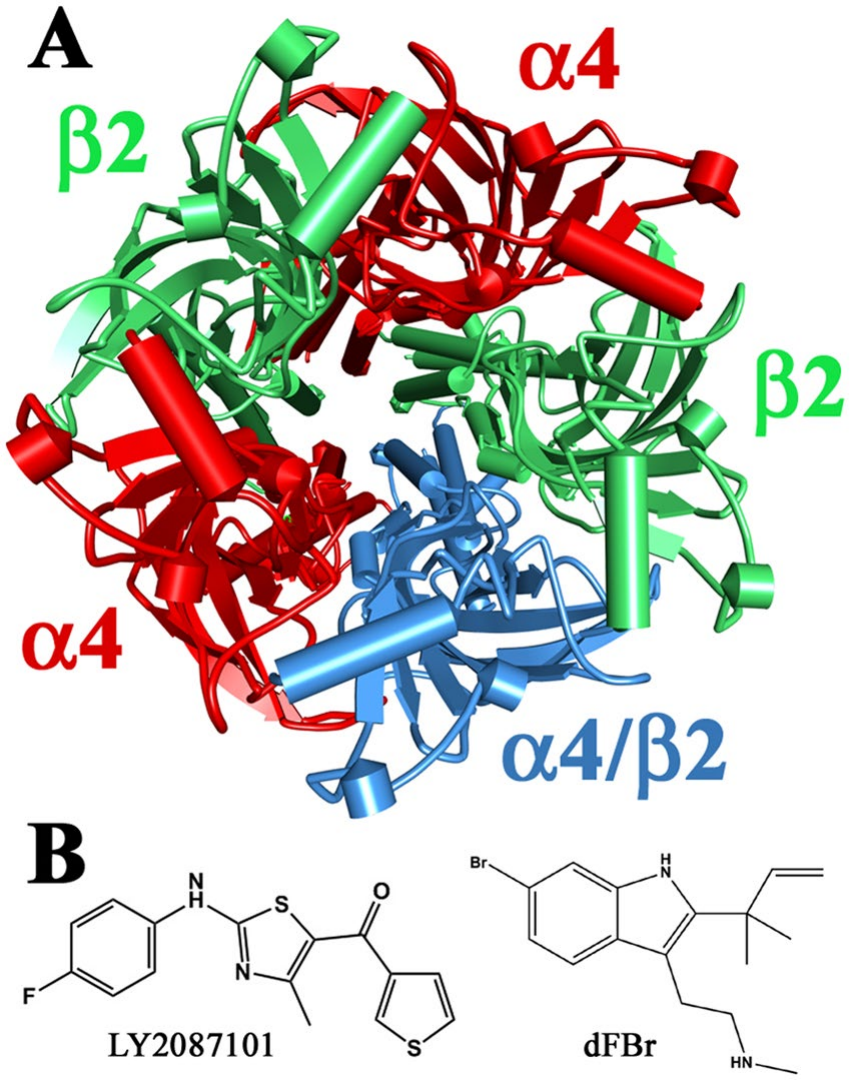
Top views depicting α4β2 nAChR based on the X-ray structure of human (α4)2(β2)3 nAChR (PDB: 5KXI) and the chemical structures of LY2087101 and dFBr.

There are nine neuronal α nAChR subunits (α2–α10) and three neuronal β nAChR subunits (β2–β4). All nAChR subunits share a general secondary structure comprising an extracellular N-terminal domain (ECD) consisting of a 10-strand β sandwich, a transmembrane domain (TMD) consisting of a loose bundle of 4 transmembrane helices (TM1–TM4), and a short extracellular C-terminus^6^. The α nAChR subunits can assemble into functional homopentameric nAChRs (e.g. α7 nAChR, the major homopentameric nAChR in the brain) or are obligate heteropentameric that can only form functional nAChRs when assembled with β subunits (e.g. α4β2, the major heteropentameric nAChR in the brain). Functional neuronal nAChRs contain two or more identical ACh binding sites (e.g. five α7:α7 ACh binding sites in the α7 nAChR and two α4:β2 ACh binding sites in the α4β2 nAChR) within the extracellular domain (ECD) at the interface of α subunit and its adjacent α or β subunit, with ACh (agonist) occupancy at least two of them are required for channel gating^1^. Because heteropentameric nAChRs can incorporate more than one type of α or β subunits within a functional receptors (e.g. α4α6β2 nAChR)^3^, they may contain more than one class of ACh binding sites. The most documented example of such ACh binding site diversity within a functional nAChR is the α4β2 nAChRs. The α4β2 nAChRs exist in two isoforms, (α4)3(β2)2 and (α4)2(β2)3 nAChRs (Fig. 1A), with the latter believed to constitute the majority of α4β2 nAChR in the cortex^7^. The (α4)3(β2)2 and (α4)2(β2)3 nAChRs contain two canonical α4:β2 ACh-binding sites that bind ACh and other agonist with high affinity (ACh EC50, ~1 μM). Additionally, the (α4)3(β2)2 contains a second class of ACh binding sites at the α4:α4 subunit interface which binds ACh with low affinity (ACh *EC*_50_, ~100 μM)^8,9^.

Enhancement of cholinergic nicotinic receptors has been shown to produce antinociceptive and anti-inflammatory effects^10^, to improve neuronal survival following cerebral ischemia and ischemic strokes^11^, to reduce the need for tobacco intake and to reverse nicotine withdrawal signs^12,13^, and to enhance cognitive functions^14–17^. In addition to agonists which bind at the ACh binding site and directly activate and gate the nAChR cationic channels, nAChR responses can be enhanced allosterically via positive allosteric modulators (PAMs) that bind at sites within the nAChR structure that are different from the agonist binding site^18^. The fact that nAChR PAMs enhance the potency and/or efficacy of endogenously release ACh is considered an inherent advantage over classical agonists which continuously activate nAChR regardless of endogenous ACh signaling level. Therefore, nAChR PAMs have drawn increasing attention as a promising treatment strategy for disorders associated with reduced cholinergic tone and/or malfunction or loss of brain nAChRs^19,20^. However, unlike agonist, less is known about the pharmacology of nAChR PAMs especially structural information pertinent to the number, location, and nAChR subtype specificity of their binding sites^21^.

Many ligands are known to act as nAChR PAMs including LY2087101 ([2-[(4-Fluorophenyl)amino]-4-methyl-5-thiazolyl]-3-thienylmethanone) and desformylflustrabromine (dFBr; N-(2-[6-bromo-2(1,1-dimethyl-2-propyl)-1H-indol-3-yl]ethyl-N-methylamine), the two compounds we study in this report. dFBr is a naturally occurring metabolite of the marine bryozoan *Flustra foliacea* that potentiates α4β2 nAChRs but not α3β2 or α7 nAChRs^22,23^. Whereas LY2087101 was developed via high-throughput screening and potentiates α4β2 and α7 nAChRs but not α3β2 nAChRs^24^. PAMs of nAChR are classified into Type I or Type II based on their effects on nAChR gating kinetics and ACh-mediated responses. Type I PAMs predominantly increase ACh sensitivity and enhance peak ACh-induced current with no effect on the kinetics of channel gating. Whereas Type II PAMs affect peak ACh-current response and the kinetics profile of channel favoring a longer open channel and decreased desensitization^18,19^. dFBr is a Type II PAM; it potentiates saturated concentrations of ACh^23,25^ and thought to alter channel gating of α4β2 nAChR increasing the frequency of channel openings and prolonging open channel duration^21,26^. LY2087101 is considered a Type I nAChR PAM because it potentiates peak agonist-evoked responses of nAChRs with little effect on the rate of receptor desensitization^27^.

Here we use the selectivity profile of LY2087101 (α4 vs. α3/5-HT3A subunits) to reveal structural information to facilitate the design of nAChR PAM. We use mutational analyses of amino acids within α4 nAChR subunit to their corresponding amino acids within the α3 nAChR and/or 5-HT3A subunit as well as homology modeling and docking routines to evaluate the binding sites of LY2087101 within the α4β2 nAChRs. Our results identified two LY2087101 binding sites within the transmembrane domain of α4β2 nAChRs; an intrasubunit binding site within the α4 subunit helix bundle at the level of α4Leu260 and α4Thr261 and an intersubunit site at the α4:α4 subunit interface above the level of α4Phe316 and extends toward the α4 extracellular end. We also show that dFBr binds at these two sites identified for LY2087101 albeit with subtle differences in amino acids contacts.

## Results

### LY2087101 potentiation of ACh-induced current of low- and high-agonist sensitivity α4β2 nAChRs

**Figure 2:**
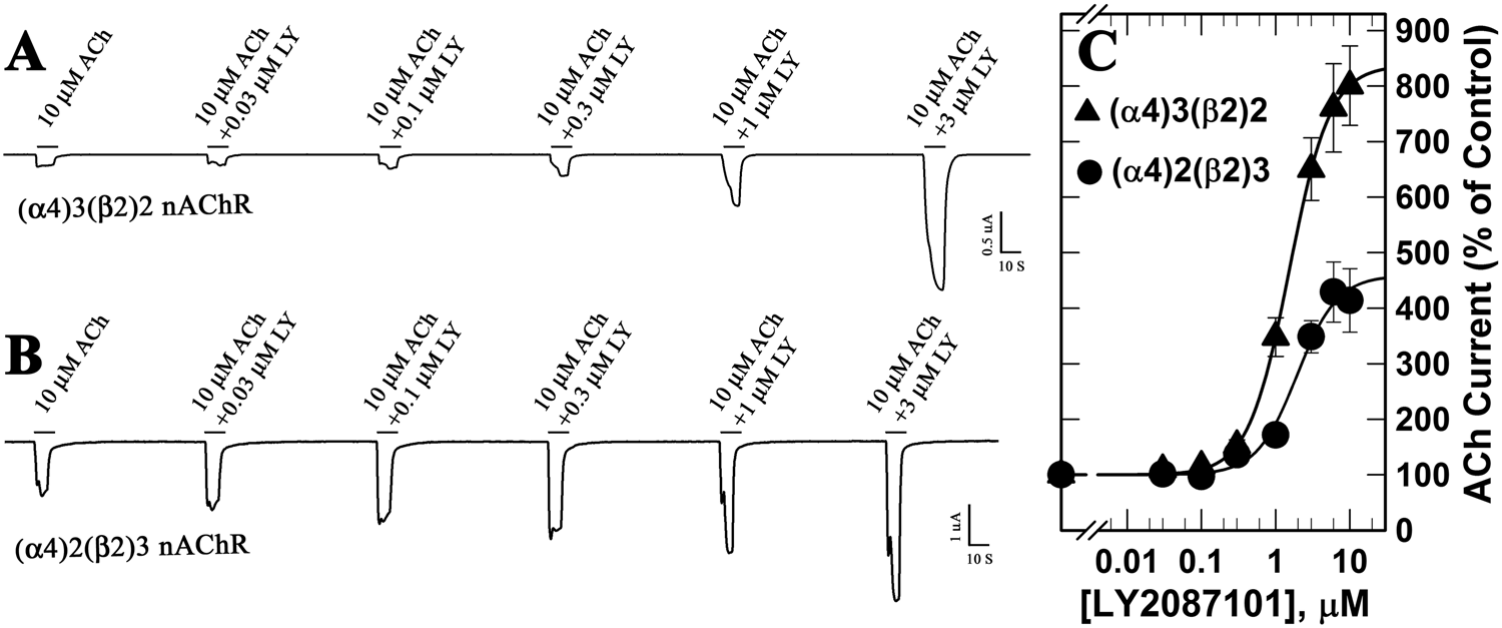
LY2087101 potentiates low- and high-sensitivity α4β2 nAChRs. *Xenopus* oocytes expressing human (α4)3(β2)2 (▲) or (α4)2(β2)3 (●) nAChRs were voltage-clamped at −50 mV, and currents elicited by 10-second applications of 10 µM ACh were recorded in the absence or presence of increasing concentration of LY2087101. (**A** and **B**) representative two-electrode voltage clamp traces showing the effect of increasing concentrations of LY2087101 on ACh-induced current responses of *Xenopus* oocytes expressing (α4)3(β2)2 and (α4)2(β2)3 nAChRs, respectively. (**C**) For each recording run, peak currents response were normalized to the peak current response elicited by 10 µM ACh alone, replicas from individual oocytes were averaged. Data (Average ± SE) from 26 (▲) and 7 (●) oocytes were plotted and fit to three parameters equation (Equation 1). LY2087101 potentiation of (α4)3(β2)2 and (α4)2(β2)3 nAChRs was characterized by *EC*_50_s of 1.4 ± 0.03 and 1.9 ± 0.04 µM, and *I*_*max*_ of 837 ± 7 and 459 ± 34%, respectively.

LY2087101 was identified as a PAM of α7 and α4β2 nAChRs in a high-throughput screen at Eli Lilly and Company^24^ and its interaction with the homopentameric α7 nAChR was characterized^27^. To begin to characterize LY2087101 interaction with the heteropentameric α4β2 nAChRs, we examined the selectivity of LY2087101 for the two α4β2 nAChR isoforms; high-agonist sensitivity (α4)2(β2)3 and low-agonist sensitivity (α4)3(β2)2 nAChRs. *Xenopus* oocytes were injected with α4 and β2 subunits RNAs at ratios 8:1 and 1:8 to favor expression of (α4)3(β2)2 and (α4)2(β2)3 nAChRs, respectively. Then the effects of LY2087101 on ACh-induced currents were examined using two-electrode voltage-clamp recording (Fig. 2). Co-application of increasing concentrations of LY2087101 with 10 μM ACh potentiated ACh responses of (α4)3(β2)2 and (α4)2(β2)3 nAChRs with similar potentiation *EC*_50_s (1.4 ± 0.03 and 1.9 ± 0.04 µM, respectively). For both (α4)3(β2)2 and (α4)2(β2)3 nAChRs, maximum LY2087101 potentiation was achieved at 10 µM LY2087101; however, the potentiation *I*_*max*_ was higher for (α4)3(β2)2 than (α4)2(β2)3 nAChR (837 ± 7 vs. 459 ± 34%).

**Figure 3:**
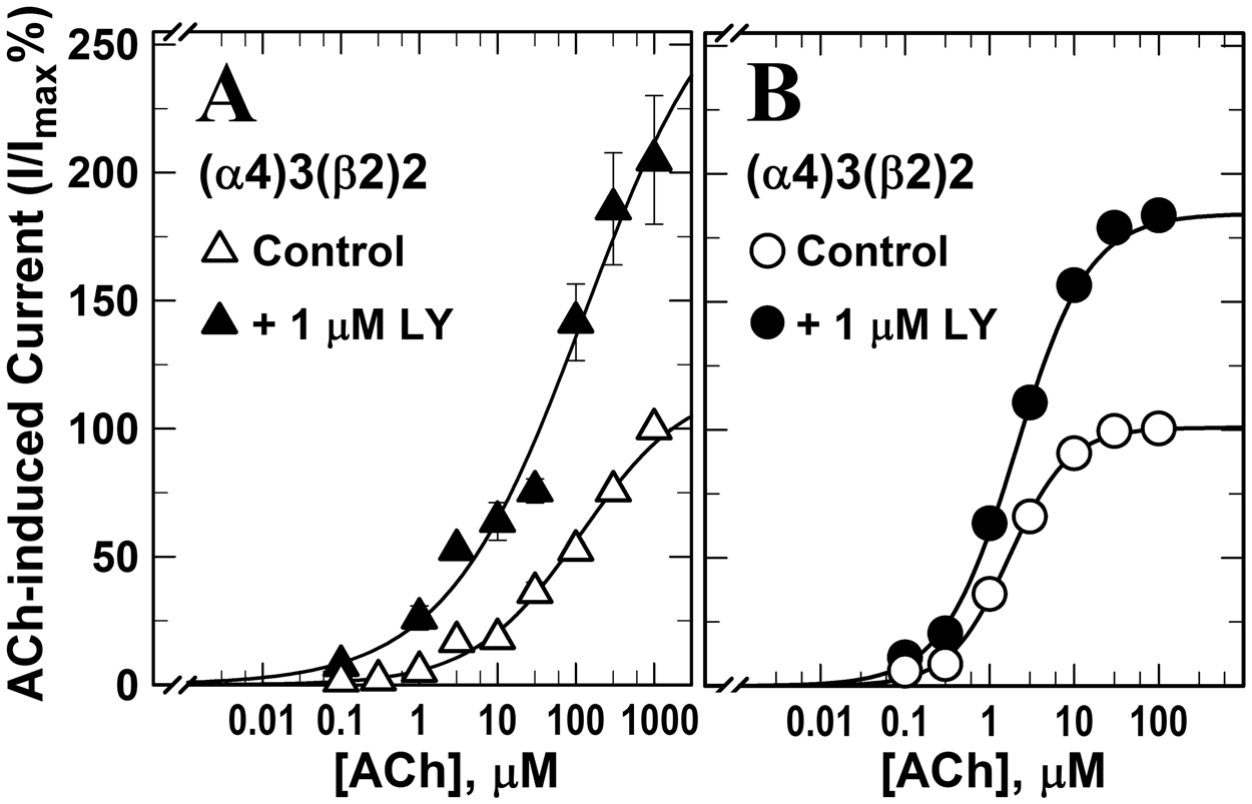
Effect of LY2087101 on the ACh dose-response curves of low- and high-sensitivity α4β2 nAChRs. ACh current responses of *Xenopus* oocytes expressing human (α4)3(β2)2 (**A**, △, ▲) or (α4)2(β2)3 (**B**, ●, ○) nAChRs were recorded in the absence of any other drug (△,○) and in the presence of 1 µM LY2087101 (▲, ●). For each ACh concentration (−/+LY2087101), peak current response was normalized to the peak current response elicited by 1 mM ACh in the same recording run. Replicas from individual oocytes were averaged and Averages ± SE for N oocytes (**A**, 18 (△), 8 (▲); **B**, 12 (○), 13 (●)) were plotted and fit to Equation 1. Values of ACh *EC*_50_/*h*/*I*_*max*_ were: for (α4)3(β2)2 nAChR (**A**), ACh (control), 113 ± 26 μM/0.66 ± 0.06/117 ± 6%; ACh(+1 LY2087101), 71 ± 25 μM/0.7 ± 0.1/251 ± 19%; for (α4)2(β2)3 nAChR (**B**), ACh (control), 1.7 ± 0.1 μM/1.2 ± 0.03/100 ± 1%; ACh (+1 LY2087101), 2.1 ± 0.1 μM/1.0 ± 0.04/189 ± 2%.

**Figure 4:**
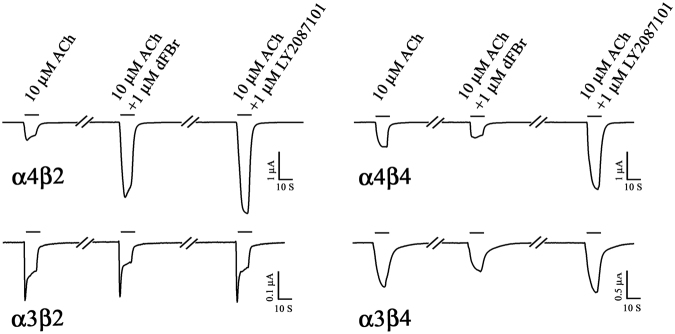
LY2087101 potentiation of α4β2 nAChR requires α4 but not β2 subunits. Representative two-electrode voltage clamp traces showing the effect of LY2087101 or dFBr at 1 μM on ACh-induced current responses of *Xenopus* oocytes expressing α4β2, α4β4, α3β2, or α3β4 nAChR.

We also examined the effect of LY2087101 on the ACh dose-response curve of (α4)3(β2)2 and (α4)2(β2)3 nAChRs (Fig. 3). We recorded current responses to increasing concentrations of ACh (0.1–1000 μM) in the absence and presence of 1 μM LY2087101. In the presence of 1 μM LY2087101, ACh maximal responses of (α4)3(β2)2 and (α4)2(β2)3 nAChRs were enhanced to 251 ± 19 and 189 ± 2%, respectively, compared with control (ACh alone) with little effect on ACh potency (ACh *EC*_50_*s* (−/+ LY2087101) were 113 ± 26/71 ± 25 and 2.1 ± 0.1/1.7 ± 0.1 μM, respectively). Next, *Xenopus* oocytes were injected with RNA mixes of α4:β2, α3:β4, α4:β2, or α4:β4 at ratios of 8:1, 1:1, or 1:8 to express nAChRs with different subunit composition and with various α:β ratios. 1 μM LY2087101 potentiated ACh responses of *Xenopus* oocytes expressing α4β2 and α4β4 but not α3β2 or α3β4 nAChR (Fig. 4) indicating that it interacts mainly with the α4 subunit. In contrast, dFBr only potentiated α4β2 nAChR and did not potentiate α4β4, α3β2, or α3β4 nAChR.

### Effect of mutations in the α4 subunit extracellular domain on modulation by LY2087101

**Figure 5:**
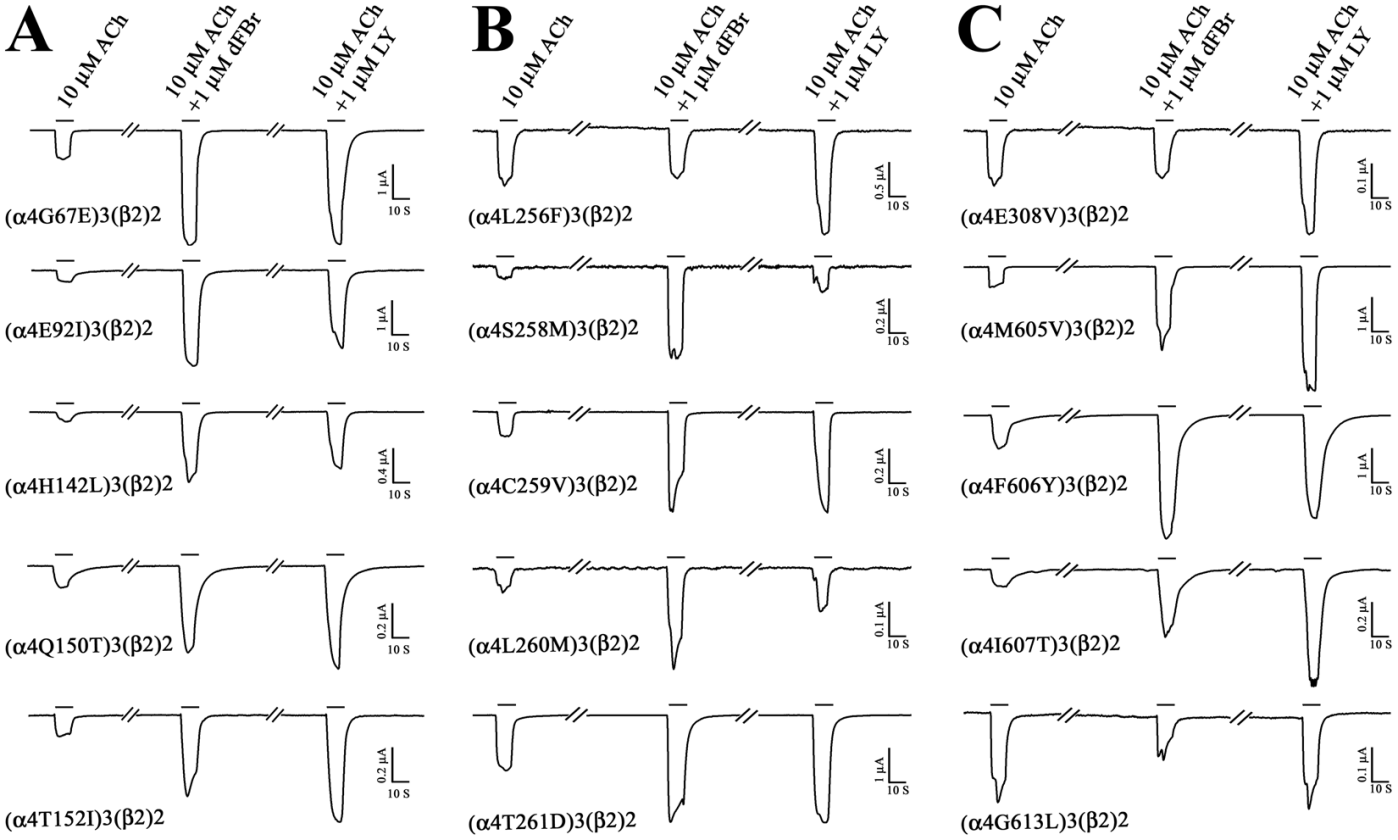
Effect of Amino acid substitutions within α4 extracellular and transmembrane domains on LY2087101 and dFBr potentiation of (α4)3(β2)2 nAChR. Representative two-electrode voltage clamp traces showing the effect of 1 μM LY2087101 or 1 μM dFBr on ACh-induced current responses of *Xenopus* oocytes expressing (α4)3(β2)2 nAChRs containing an amino acid substitution at the α4 subunit extracellular domain (**A**) or within the α4 subunit transmembrane domain (**B** and **C**).

Results shown in Fig. 4 and the fact that LY2087101 potentiates (α4)3(β2)2 to a higher *Imax* than (α4)2(β2)3 nAChR establish that α4 is sufficient to confer LY2087101 binding and modulation of nAChR channel gating. Therefore, we tested the possibility that LY2087101 shares same determinants for potentiation with other nAChR PAMs (NS9283 and CMPI) that require the presence of α4 for nAChR modulation. Amino acid substitution within the extracellular domain have been shown to reduce (α4)3(β2)2 nAChR potentiation by CMPI and NS9283. Previous work^28,29^ has shown that amino acid substitutions at α4His142 (*numbering start from the translational N-terminus of α4 subunit, subtract 26 amino acid to get numbering based on the recently published crystal structure of (α4)2(β2)3 nAChR (PDB# 5KXI*)^6^ selectively reduces potentiation by NS9283, while amino acid substitutions at α4Gly67, α4Lys90, and α4Glu92 selectively reduce potentiation by CMPI and at α4Gln150 and α4Thr152 reduce potentiation by CMPI and NS9283. The effects of amino acid substitutions at these positions on LY20871010 potentiation were assessed by recording ACh-induced current responses (±1 μM LY208710 or 1 μM dFBr) of *Xenopus* oocytes injected with a mix of RNA encoding α4 containing these point mutations and WT β2 subunit RNA at a ratio of 8:1 to favor the expression of the 3α4:2β2 stoichiometry. As shown in Fig. 5A, mutations of these amino acids had no effects on LY2087101 potentiation of (α4)3(β2)2 nAChR. To quantify the effect of amino acid mutations on LY2087101 potentiation, we calculated for WT and for each mutation a potentiation ratio ***PR***, the ratio of the peak current amplitude induced by an agonist at its EC_10_ (10 µM ACh for (α4)2(β2)3 nAChR) in the presence of 1 μM PAM (LY2087101 or dFBr) relative to the peak current amplitude elicited by the agonist at its EC_10_ alone (Table 1). LY2087101 potentiation ratios for (α4)3(β2)2 nAChRs containing point mutations within the α4 extracellular domain mutation to the corresponding amino acids in the α3 subunit (α4L63I, α4R65H, α4G67E, α4E92I, α4H94N, α4H142L, α4R148E, α4Q150T, or α4T152I) were similar or higher than that for WT (α4)3(β2)2 nAChR (***PR***_*WT*_ = 4.1 ± 0.3) and significantly different from no potentiation (***PR*** = *1*) with a *P* values of <0.001 in one-way analyses of variance (ANOVA) test. These results indicate that the extracellular domain of α4 subunit has little, if any, direct interaction with LY2087101.

**Table 1:** Modulation of WT and mutants (α4)3(β2)2 nAChRs by LY2087101 and dFBr.

Combination	5KXI α4 numbering	*R*_(LY2087101)_			*R*_(dFBr)_		
		Ave ± SE	N	P	Ave ± SE	N	P
(α4)3(β2)2	WT	4.1 ± 0.3	47	<0.001	4.4 ± 0.2	27	<0.001
(α4L63I)3(β2)2	α4L37I	4.3 ± 0.4	5	<0.001	3.5 ± 0.3^a^	5	<0.001
(α4R65H)3(β2)2	α4R39H	7.4 ± 1.3	3	<0.001	5.3 ± 1.8^a^	3	<0.001
(α4G67E)3(β2)2	α4G41E	5.2 ± 0.7	6	<0.001	4.1 ± 0.2^a^	6	<0.001
(α4E92I)3(β2)2	α4E66I	10.9 ± 2.6	3	<0.001	9.3 ± 1.8^a^	7	<0.001
(α4H94N)3(β2)2	α4H68N	5.2 ± 1.0	3	<0.001	5.8 ± 0.5^a^	7	<0.001
(α4H142L)3(β2)2	α4H116L	8.7 ± 0.5	3	<0.001	8.1 ± 0.6^a^	5	<0.001
(α4R148E)3(β2)2	α4R122E	11.1 ± 1.2	5	<0.001	10.3 ± 2.4^a^	5	<0.001
(α4Q150T)3(β2)2	α4Q124T	4.3 ± 0.2	6	<0.001	3.9 ± 0.1^a^	6	<0.001
(α4T152I)3(β2)2	α4T126I	4.3 ± 1.3	6	<0.001	2.6 ± 0.4^a^	8	0.004
(α4C254S)3(β2)2	α4C228S	8.4 ± 1.5	11	<0.001	3.6 ± 0.4	9	<0.001
(α4L256F)3(β2)2	α4L230F	1.0 ± 0.1	10	1.000	1.0 ± 0.1	10	1.000
(α4S258M)3(β2)2	α4S232M	1.5 ± 0.2	16	0.322	5.0 ± 0.5	12	<0.001
(α4C259F)3(β2)2	α4C233F	3.2 ± 0.4	7	0.002	3.1 ± 0.3	5	0.002
(α4C259V)3(β2)2	α4C233V	5.2 ± 0.6	10	<0.001	4.6 ± 0.9	3	<0.001
(α4L260M)3(β2)2	α4L234M	1.6 ± 0.2	18	0.215	3.6 ± 0.4	15	<0.001
(α4T261D)3(β2)2	α4T235D	1.6 ± 0.1	18	0.215	2.1 ± 0.1	9	0.045
(α4I275V)3(β2)2	α4I249V	3.7 ± 0.5	8	<0.001	4.0 ± 0.6	5	<0.001
(α4S284G)3(β2)2	α4S258G	3.8 ± 0.4	6	<0.001	2.8 ± 0.4	3	0.035
(α4L291V)3(β2)2	α4L265V	3.7 ± 0.6	12	<0.001	3.5 ± 0.2	7	<0.001
(α4E308V)3(β2)2	α4E282V	1.7 ± 0.2	8	0.294	1.0 ± 0.1	8	1.000
(α4L310F)3(β2)2	α4L284F	9.8 ± 1.0	7	<0.001	5.6 ± 0.3	2	<0.001
(α4L311V)3(β2)2	α4L285V	15.6 ± 3.2	3	<0.001	5.8 ± 1.4	3	<0.001
(α4F312V)3(β2)2	α4F286V	2.5 ± 0.2	6	0.048	1.2 ± 0.1	7	0.738
(α4T313C)3(β2)2	α4T287V	2.1 ± 0.2	4	0.226	3.9 ± 0.4	4	<0.001
(α4I315A)3(β2)2	α4I289A	2.5 ± 0.2	6	0.048	1.2 ± 0.2	3	0.814
(α4F316L)3(β2)2	α4F290L	1.1 ± 0.1	10	0.869	1.2 ± 0.1	10	0.703
(α4H332Y)3(β2)2	α4H306Y	4.2 ± 0.5	10	<0.001	5.5 ± 0.4	6	<0.001
(α4M605I)3 (β2)2	α4M364I	4.5 ± 0.2	11	<0.001	4.5 ± 0.3	6	<0.001
(α4M605V)3(β2)2	α4M364V	3.8 ± 1.1	6	<0.001	5.3 ± 1.2	3	<0.001
(α4F606Y)3(β2)2	α4F365Y	2.3 ± 0.3	10	0.033	3.1 ± 0.4	6	0.001
(α4I607T)3(β2)2	α4I366T	5.8 ± 0.7	6	<0.001	4.7 ± 2.1	3	<0.001
(α4I608L)3(β2)2	α4I367L	4.4 ± 0.6	7	<0.001	5.4 ± 0.4	4	<0.001
(α4V609A)3(β2)2	α4V368A	5.7 ± 1.1	7	<0.001	3.2 ± 0.2	4	<0.001
(α4G613L)3(β2)2	α4G372L	1.09 ± 0.1	9	0.887	0.8 ± 0.1	8	0.725

Current responses to 10 µM ACh, 10 µM ACh + 1 µM LY2087101, and 10 µM ACh + 1 µM dFBr were recorded from oocytes expressing WT and mutants (α4)3(β2)2 nAChRs (Fig. 5). ***R***, the ratio of peak current amplitude in the presence of 1 µM PAM relative to peak current amplitude elicited by 10 µM ACh alone from the same recording run were calculated. Replicas from the same oocyte were averaged. The data in the table are Average ± SE of several oocytes (N). The probability (P) that calculated ***R*** differs from no potentiation (***R*** = 1) was analyzed using one-way analysis of variance with the Holm-Sidak Test (SigmaPlot, Systat Software Inc.). “^a^”Indicates data reported previously^29^.

### Effect of mutations in the α4 subunit transmembrane domain on modulation by LY2087101

Because LY2087101 does not potentiate α3-containing nAChRs or the serotonin (5-HT3) receptor we reasoned that point mutations within the transmembrane domains making the α4 subunit more similar to the α3 or 5-HT3-A subunit will lead to identification of amino acid residues that confer LY2087101 potentiation in the (α4)3(β2)2 nAChR. A total of 23 amino acid residues (6 within TM1, 3 within TM2, 8 within the TM3, and 6 within the TM4) were identified through sequence alignments of α4 subunit with and α3 subunits and 5-HT3A subunit (Supplementary Figure 1) and substituted to the corresponding amino acids within the α3 nAChR and/or 5-HT3A subunit. The effects of these amino acid mutations on LY2087101 potentiation were assessed by recording ACh-induced current responses (±1 μM LY2087101 or 1 μM dFBr) of *Xenopus* oocytes expressing (α4)3(β2)2 nAChR containing these point mutations (Fig. 5B,C) and the LY2087101 potentiation ratios were quantified (Table 1; Supplementary Figure 2). For (α4)3(β2)2 nAChRs containing α4C254S, α4C259F, α4C259V, α4I275V, α4S284G, α4L291V, α4L310F, α4L311V, α4H332Y, α4M605I, α4M605V, α4I607T, α4I608L, or α4V609A mutation, LY2087101 ***PRs*** were significantly different from no potentiation. In contrast, LY2087101 ***PRs*** for (α4)3(β2)2 nAChRs containing α4L256F, α4S258M, α4L260M, α4T261D, α4E308V, α4F316L, or α4G613L mutation were reduced to <1.7 fold and were non-significantly different from no potentiation with *P* values of >0.2 in one-way ANOVA (Table 1). These results establish a selective role of α4Leu256, α4Ser258, α4Leu260, α4Thr261, α4Glu308, α4Phe316, and α4Gly613 in LY2087101 potentiation of the (α4)3(β2)2 nAChR. LY2087101 ***PRs*** for α4F312V, α4T313C, α4I315A, and α4F606Y were 2.1–2.5 revealing a reduced LY2087101 potentiation compared with wild type. This indicates that α4Phe312, α4Thr313, α4I315A, and α4Phe606 contribute to but are not absolutely required for LY2087101 potentiation of (α4)3(β2)2 nAChR.

**Figure 6:**
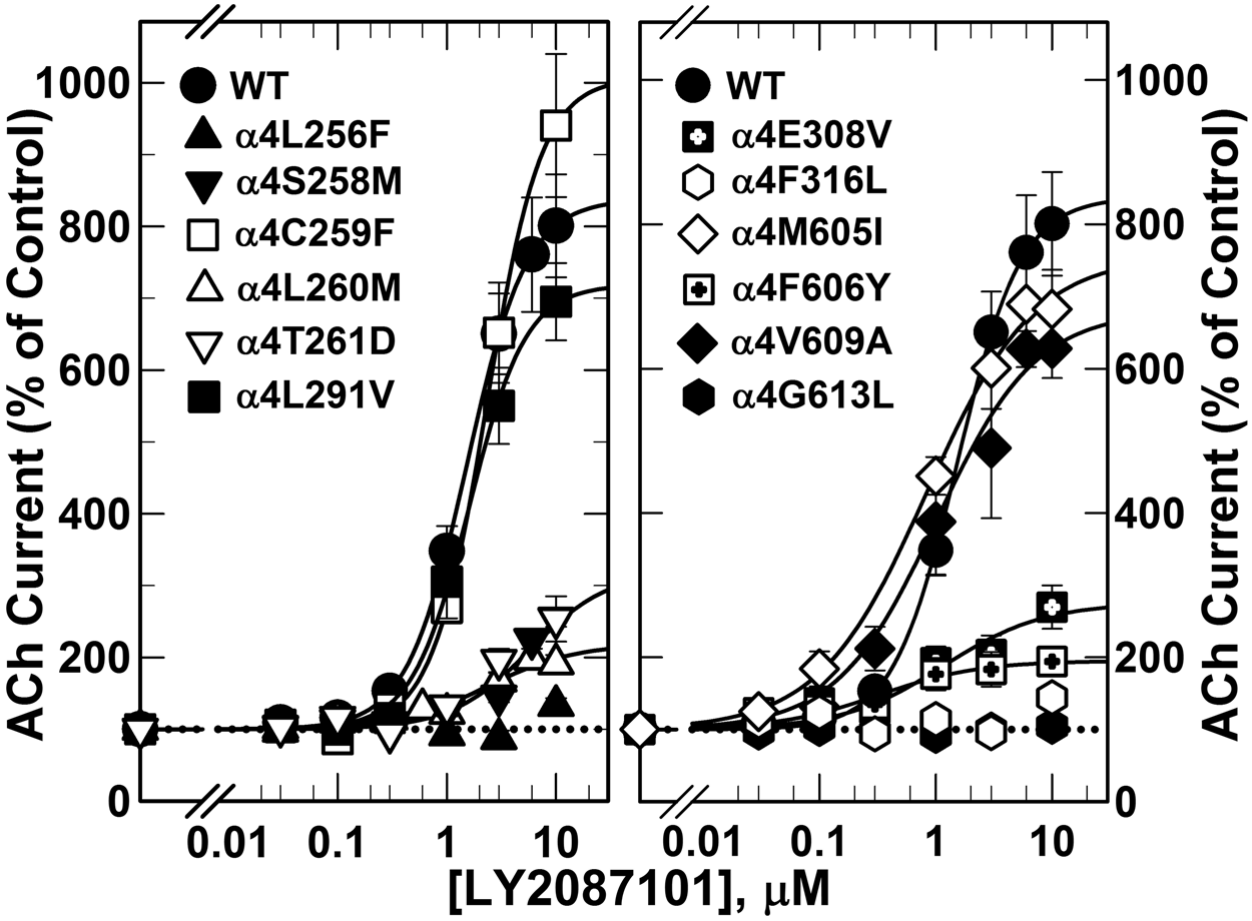
LY2087101 concentration-dependent potentiation of WT and mutant α4β2 nAChRs. Peak ACh-induced current responses of *Xenopus* oocytes expressing WT or mutant (α4)3(β2)2 nAChRs in the presence of increasing concentrations of LY2087101 were normalized to the peak current elicited by 10 µM ACh alone. Shown are data (Average ± SE) from several oocytes. Data were fit to a single site model using Equation 1 and parameters (potentiation *EC*_50_ and *I*_*max*_) are shown in Table 2.

To define the LY2087101 concentration-dependent potentiation of (α4)3(β2)2 nAChR containing amino acid substitution within the α4 subunit transmembrane domain, current responses to 10 µM ACh (*EC*_10_) alone or in the presence of increasing concentrations of LY2087101 on oocytes expressing (α4)3(β2)2 nAChRs containing point mutations were recorded (Fig. 6). The observed current responses for oocytes expressing (α4)3(β2)2 nAChRs containing α4L256F, α4F316L, or α4G613L mutation to 10 µM ACh in the presence of LY2087101 at any concentration tested (0.03–10 µM) was not significantly different from 10 µM ACh alone with *P* values of >0.5 in one-way ANOVA test (Table 2). For oocytes expressing (α4)3(β2)2 nAChRs containing α4S258M, α4L260M, α4T261D, α4E308V, α4F312V, α4T313C, α4I315A, or α4F606Y mutation, potentiation *Imax* at 10 µM LY2087101 were 254 ± 12, 269 ± 21, 215 ± 29, 270 ± 30, 269 ± 43, 196 ± 23, 405 ± 5, and 195 ± 13% which significantly reduced (*P* < 0.001 in one-way ANOVA; data not shown) compared with WT (α4)3(β2)2 nAChR (LY2087101 potentiation *I*_*max*_ = 837 ± 7%). For (α4)3(β2)2 nAChRs containing α4V609A mutation, potentiation *I*_*max*_ was >500% and significantly (P < 0.001) different from 10 µM ACh alone with *P* values of <0.001 in one-way ANOVA test.

**Table 2:** LY2087101modulation of WT and mutants (α4)3(β2)2 nAChRs.

Combination	5KXI α4 numbering	EC50(μM)	Imax%	N	P
(α4)3(β2)2 WT	LS WT	1.5 ± 0.03	837 ± 7	26	<0.001
(α4)2(β2)3 WT	HS WT	1.9 ± 0.04	459 ± 34	7	<0.001
(α4C254S)3(β2)2	α4C228S	1.7 ± 0.2	1058 ± 43	5	<0.001
(α4L256F)3(β2)2	α4L230F	ND	133 ± 10*	4	0.600
(α4S258M)3(β2)2	α4S232M	ND	254 ± 12*	6	0.004
(α4L260M)3(β2)2	α4L234M	2.5 ± 0.6	269 ± 21	3	0.019
(α4T261D)3(β2)2	α4T235D	2.4 ± 1.6	215 ± 29	9	0.012
(α4I275V)3(β2)2	α4I249V	1.7 ± 0.2	1029 ± 51	3	<0.001
(α4S284G)3(β2)2	α4S258G	0.7 ± 0.1	537 ± 20	4	<0.001
(α4L291V)3(β2)2	α4L265V	1.6 ± 0.1	719 ± 20	9	<0.001
(α4E308V)3(β2)2	α4E282V	ND	270 ± 30*	5	0.003
(α4L310F)3(β2)2	α4L284	3.1 ± 0.1	3617 ± 42	5	<0.001
(α4F312V)3(β2)2	α4F286V	0.1 ± 0.01	269 ± 43*	3	0.019
(α4T313C)3(β2)2	α4T287C	0.7 ± 0.1	196 ± 23	5	0.095
(α4I315A)3(β2)2	α4I289A	1.2 ± 0.5	405 ± 5	4	<0.001
(α4F316L)3(β2)2	α4F290L	ND	144 ± 2*	3	0.538
(α4H332Y)3(β2)2	α4H306Y	1.3 ± 0.1	573 ± 20	4	<0.001
(α4M605I)3(β2)2	α4M364I	0.8 ± 0.1	756 ± 27	5	<0.001
(α4M605V)3(β2)2	α4M364V	2.7 ± 0.02	856 ± 3	3	<0.001
(α4F606Y)3(β2)2	α4F365Y	0.3 ± 0.2	195 ± 13	4	0.133
(α4I607T)3(β2)2	α4I336T	0.8 ± 0.1	958 ± 32	3	<0.001
(α4I608L)3(β2)2	α4I367L	1.2 ± 0.1	840 ± 31	3	<0.001
(α4V609A)3(β2)2	α4V368A	1.0 ± 0.3	680 ± 58	4	<0.001
(α4G613L)3(β2)2	α4G372L	ND	105 ± 6*	4	0.937

Current responses to 10 µM ACh alone or in the presence of increasing concentrations of LY2087101 were recorded from oocytes expressing WT and mutants (α4)3(β2)2 nAChRs. For each application, peak current amplitude was quantified and normalized to peak current amplitude elicited by 10 µM ACh alone within the same recording run. Replicas from the same oocyte were averaged, and for each LY2087101 concentration (Average ± SE) of data from several oocytes (N) were plotted (Fig. 6) and fit to equation 1. The probability (P) that an *I*_*max*_ differs from no potentiation (*I*_*max*_ = 100) was analyzed using a one-way analysis of variance with the Holm-Sidak Test. Curve fitting, parameters calculation, and statistics were performed in SigmaPlot 11 (Systat Software Inc.). *Values are not derived from curve fitting, they represent the maximum *I*_*max*_ seen for that mutant at any concentration up to 10 µM LY2087101. ND = not determined.

**Figure 7:**
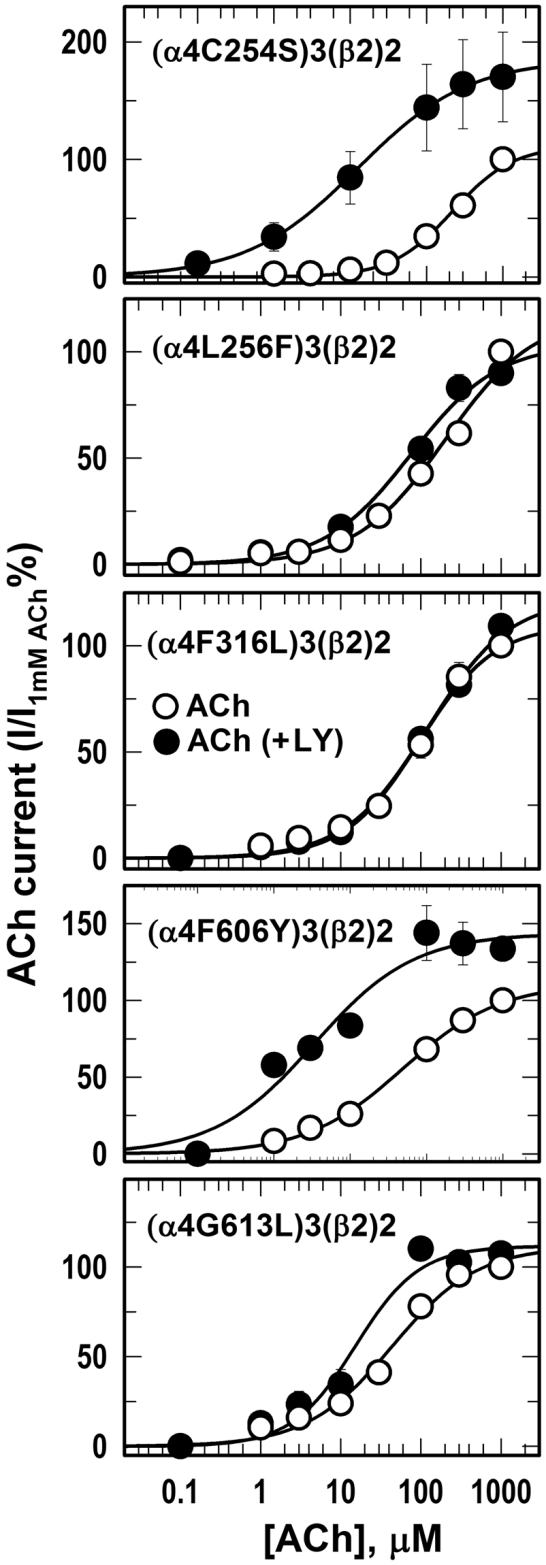
Effect of LY2087101 on the ACh dose-response curve of mutant α4β2 nAChRs. Currents elicited by *Xenopus* oocytes expressing (α4)_3_(β2)_2_ nAChRs containing point mutation within the α4 subunit in response to 10-second applications of increasing concentrations of ACh (alone (○) or +1 µM LY2087101 (●)) were recorded and normalized to peak currents elicited by 1 mM ACh alone. Replicas from the same oocyte were averaged and the Average ± SE of data from at least 3 oocytes were plotted and fit to a single site model using Equation 1. For (α4C254S)3(β2)2; (α4L256F)3(β2)2; (α4S258M)3(β2)2; (α4L260M)3(β2)2; (α4T261D)3(β2)2; (α4F316L)3(β2)2; (α4F606Y)3(β2)2; (α4G613L)3(β2)2 nAChRs, ACh *I*_*max*_ (Ave ± SEM) calculated from the fit of ACh concentration-response curves in the presence of 1 µM LY2087101 were 185 ± 04; 104 ± 09; 232 ± 10; 264 ± 80; 180 ± 23; 122 ± 13; 144 ± 013; 112 ± 9%, and the probability (*P*) that Imax in the presence of 1 µM LY2087101 were different from normalized current response to 1 mM ACh alone (*I*_*max*_ = 100) were <0.001, 0.878, <0.001, <0.001, 0.003, 0.475, 0.062, and 0.442, respectively.

We also characterized ACh concentration-responses curves for oocytes expressing mutant (α4)3(β2)2 nAChRs in the absence and presence of LY2087101 by recording current responses to increasing concentrations of ACh, alone and in the presence of 1 µM LY2087101 (Fig. 7). In WT (α4)3(β2)2 nAChR, co-application of 1 µM LY2087101 produced a shift of the ACh concentration-response curve to a higher *I*_*max*_ (~260%) with little effect on the ACh potency (Fig. 3A). For (α4)3(β2)2 nAChRs containing a point mutation at α4L256F, α4F606Y, α4F316L, or α4G613L, LY2087101 did not significantly alter the ACh *I*_*max*_ when co-applied with ACh (*I*_*max*_ ACh + 1 µM LY2087101 were 104 ± 09, 144 ± 13, 122 ± 13, and 112 ± 9%, respectively). ACh *I*_*max*_ calculated from the fit of ACh concentration-response curve in the presence of 1 µM LY2087101 and the probability (*P*) using one way ANOVA that *I*_*max*_ in the presence of 1 µM LY2087101 were different from *I*_*max*_ = 100 for other mutants examined are listed in the legend of Fig. 7.

The effect of these mutations on the LY2087101 concentration-dependent potentiation and the ACh concentration-response curve of (α4)3(β2)2 nAChR are consistent with the calculated LY2087101 ***PRs*** and further support a fundamental role of α4Leu256, α4Phe316, and α4Gly613 as well as a supporting role of α4Ser258, α4Leu260, α4Thr261, α4Glu308, α4Phe312, α4Thr313, α4Ile315, or α4Phe606, in LY2087101 potentiation of the (α4)3(β2)2 nAChR.

### Effect of mutations in the α4 subunit on modulation by desformylflustrabromine (dFBr)

dFBr is a naturally occurring metabolite of the marine bryozoan *Flustra foliacea* that potentiates ACh-induced responses of α4β2 nAChRs but not α3β2 or α7 nAChRs (Sala *et al*.^22^; Kim *et al*.^23^). dFBr potentiates both (α4)2(β2)3 and (α4)3(β2)2 nAChRs isoform with similar potentiation *EC*_50_*s* of ~1 µM and maximally by 300 and 400%, respectively^25,26^. The effect of dFBr on the ACh concentration-response curves of (α4)3(β2)2 and (α4)2(β2)3 nAChRs was characterized by ~4 fold increase in ACh efficacy (*Imax*) with little effects on ACh potency (*EC*_50_). In animal model, dFBr has been shown to reduce nicotine self-administration, to potentiate the antiallodynic response of nicotine, to reverse nicotine withdrawal signs and to attenuate compulsive-like behavior in a non-induced compulsive-like mouse model^12,29–31^.

**Figure 8:**
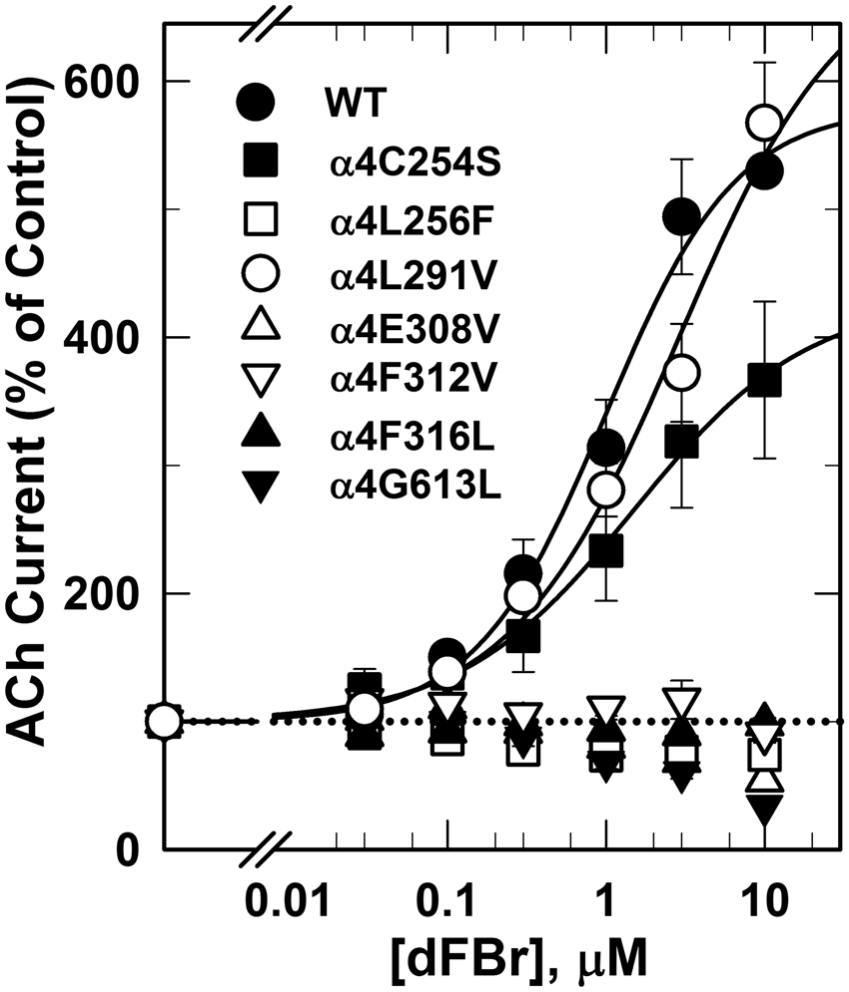
dFBr concentration-dependent potentiation of WT and mutant α4β2 nAChRs. Peak current of Xenopus oocytes expressing WT or mutant (α4)3(β2)2 nAChRs responses to 10 s application of 10 μM ACh in the absence or presence of increasing concentrations of dFBr were normalized to the peak current elicited by 10 µM ACh alone. Shown are data (Average ± SE) from at least 4 different oocytes. Data were fit to a single site model using Equation 1. Values of dFBr potentiation *EC*_*50*_/*I*_*max*_/h were: for WT (α4)3(β2)2, 1.0 ± 0.3 μM/580 ± 42%/1.1 ± 0.2; for (α4C254S)3(β2)2, 1.5 ± 0.5 μM/430 ± 31%/0.8 ± 0.1; and for (α4L291V)3(β2)2, 2.9 ± 1.9 μM/600 ± 136%/0.8 ± 0.2. For (α4L256F)3(β2)2, (α4E308V)3(β2)2, and (α4G613L)3(β2)2, inhibition at 10 μM dFBr were ~25, 45, and 65%, respectively.

**Figure 9:**
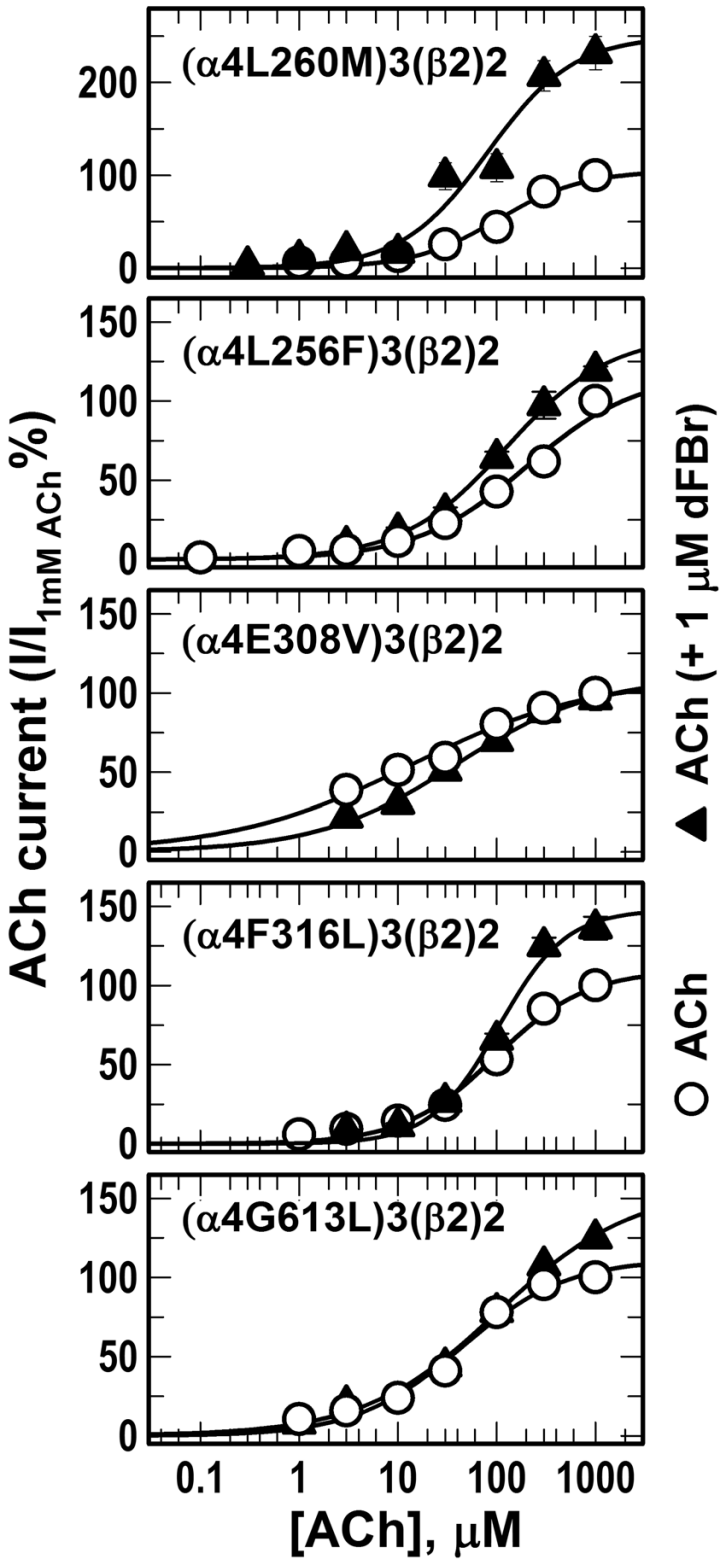
Effect of dFBr on the ACh dose-response curve of mutant α4β2 nAChRs. Currents elicited by *Xenopus* oocytes expressing mutant (α4)_3_(β2)_2_ nAChRs in response to 10-second applications of increasing concentrations of ACh (alone (○) or +1 µM dFBr (▲)) were recorded and normalized to peak currents elicited by 1 mM ACh alone. Replicas from the same oocyte were averaged and the Average ± SE of at least 3 oocytes were plotted and fit to a single site model using Equation 1. ACh *I*_*max*_ in the presence of 1 µM dFBr calculated from curve fitting for (α4C254S)3 (β2)2, (α4L256F)3 (β2)2, (α4L260M)3 (β2)2, (α4E308V)3 (β2)2, (α4F316L)3 (β2)2, (α4F606Y)3 (β2)2, (α4G613L)3(β2)2 were 190 ± 08, 142 ± 08, 250 ± 36, 110 ± 06, 148 ± 12, 159 ± 07, and 157 ± 19%, and the probability (*P*) that Imax in the presence of 1 µM dFBr were different from normalized current response to 1 mM ACh alone (*Imax* = 100) were <0.001, 0.057, <0.001, 0.602, 0.031, 0.001, 0.012, respectively.

Despite its promising clinical importance, the binding site of dFBr in the α4β2 nAChR is still under ongoing investigation. In earlier work we have shown^32^ that dFBr binds within the *Torpedo* nAChR extracellular domain at binding sites identified previously for the non-selective nAChR PAMs, galantamine and physostigmine^33^. Mutational analyses of amino acids within the extracellular domain of the β2 subunit significantly reduce dFBr potency in (α4)3(β2)2 and (α4)2(β2)3 nAChRs suggesting the involvement of amino acid projecting to β2:α4 extracellular interface in dFBr modulation of ACh-induced responses^34^. In addition, alanine substitution and substituted cysteine accessibility within the upper part of the transmembrane domain, the Cys loop within the extracellular domain and post TM4 extracellular domain demonstrated the presence of dFBr binding site within the upper half of a cavity between TM3 and TM4^35^. Furthermore, mutational analyses of amino acids projecting to the α4:α4 extracellular interface which only exist in the (α4)3(β2)2 nAChRs did not significantly alter dFBr potentiation^29^. In this study, parallel to studying LY2087101, we examined the effect of amino acid mutations within the transmembrane domain on dFBr potentiation of (α4)3(β2)2 nAChRs. dFBr potentiation ratios (***PR***, peak current amplitude of oocytes expressing WT and mutants (α4)3(β2)2 nAChRs in response to application of 10 µM ACh + 1 µM dFBr relative to peak current amplitude elicited by 10 µM ACh alone) are listed in Table 1 and shown in comparison to **PR**s calculated for LY2087101 in Supplementary Figure 2. Table 1 also includes the probability (*P*) that calculated ***PR*** differs from no potentiation (***PR*** = 1). dFBr potentiation ratios for 6 mutations within the transmembrane domain were not significantly different (*P* > 0.7 in one way ANOVA) from no potentiation (***PR***_L256F_, ***PR***_E308V_, ***PR***_F312V_, ***PR***_I315A_, ***PR***_F316L_, and ***PR***_G613L_ were 1.0, 1.0, 1.2, 1.2, 1.2, and 0.8, respectively) and were significantly different (*P* < 0.001 in one way ANOVA) from dFBr potentiation ratios for WT (α4)3(β2)2 nAChR (***PR***_*WT*_ = 4.4 ± 0.2). dFBr potentiation ratios for other mutations tested were significantly different from no potentiation (***PR*** > 3 and P < 0.005) with the exception of (α4T261D)3(β2)2 (***PR***_T261D_ = 2.1 ± 0.1; *P* = 0.045) and (α4S284G)3(β2)2 (***PR***_S284G_ = 2.8 ± 0.4; *P* = 0.035). These results indicate that amino acids α4L256, α4E308, α4F312, α4I315, α4F316, and α4G613L play a role in dFBr recognition and potentiation of (α4)3(β2)2 nAChR. To further confirm this conclusion, we tested the concentration-dependence effect of dFBr on ACh-current responses (Fig. 8) and the effect of dFBr on ACh concentration-response curves (Fig. 9) of *Xenopus* oocytes expressing (α4)3(β2)2 nAChRs containing these point mutations in comparison with that for WT (α4)3(β2)2 nAChRs. In agreement with the calculated dFBr potentiation ratios, dFBr up to 10 µM did not potentiate (α4L256F)3(β2)2, (α4E308V)3(β2)2, (α4F312V)3(β2)2, (α4F316L)3(β2)2, or (α4G613L)3(β2)2 instead inhibition of ACh-current responses of (α4L256F)3(β2)2, (α4E308V)3(β2)2, and (α4G613L)3(β2)2 nAChRs were observed at 10 μM dFBr (Fig. 8). Similarly, ACh concentration-response curve of (α4L256F)3(β2)2, (α4E308V)3(β2)2, (α4F312V)3(β2)2, (α4F316L)3(β2)2, or (α4G613L)3(β2)2 recorded in the presence of 1 μM dFBr were not significantly different from control ACh concentration response curves (Fig. 9**)** and *Imax* calculated from curve fitting in the presence of 1 μM dFBr were 142 ± 08, 110 ± 06, 148 ± 12, 159 ± 07, and 157 ± 19% for (α4L256F)3(β2)2, (α4E308V)3(β2)2, (α4F316L)3(β2)2, (α4F606Y)3(β2)2, and (α4G613L)3(β2)2, respectively.

## Discussion

There is a clinical need to selectively target neuronal nicotinic acetylcholine receptors (nAChRs). Positive allosteric modulators (PAMs) represent, in principle, a promising strategy to fulfill this need while avoiding non-physiological alteration in cholinergic tone and side effects associated with binding at multiple nAChRs subtypes. LY2087101, the compound we study here, is a unique nAChR PAM as it potentiates α7 and α4-containing nAChRs but not α3-containing nAChRs. We are using the selectivity profile of LY2087101 (α4 vs. α3 subunits) to reveal structural information that would facilitate the design of nAChR PAMs with higher nAChR subunit selectivity. We focus on LY2087101 interaction with the α4β2 nAChRs, the most abundant heteropentameric nAChRs in the human brain, using *Xenopus* oocytes expression system, site-directed mutagenesis, and two-electrode voltage-clamp electrophysiological recordings. This technique is very instrumental in studying drug interactions with ligand-gated ion channels such as the nAChRs and in unraveling the role of certain amino acid residues in these interactions. We found that co-application of increasing concentrations of LY2087101 with 10 μM ACh potentiated ACh responses of (α4)3(β2)2 and (α4)2(β2)3 nAChRs with similar potencies (*EC*50*s*, 1–2 μM) albeit with higher efficacy at (α4)3(β2)2 than (α4)2(β2)3 nAChR (*Imax*, ~840 and 460%, respectively; Fig. 2). LY2087101 increased ACh maximal responses of (α4)3(β2)2 and (α4)2(β2)3 nAChRs (~250 and 190%, respectively) with little effect on the potency of ACh (Fig. 3). LY2087101 potentiated ACh responses of α4β2 and α4β4 but not α3β2 or α3β4 nAChR indicating that it interacts mainly with the α4 subunit (Fig. 4) indicating that α4 subunit is required and sufficient to confer LY2087101 binding and potentiation.

### LY2087101 binding sites in the (α4)3(β2)2 nAChR

**Figure 10:**
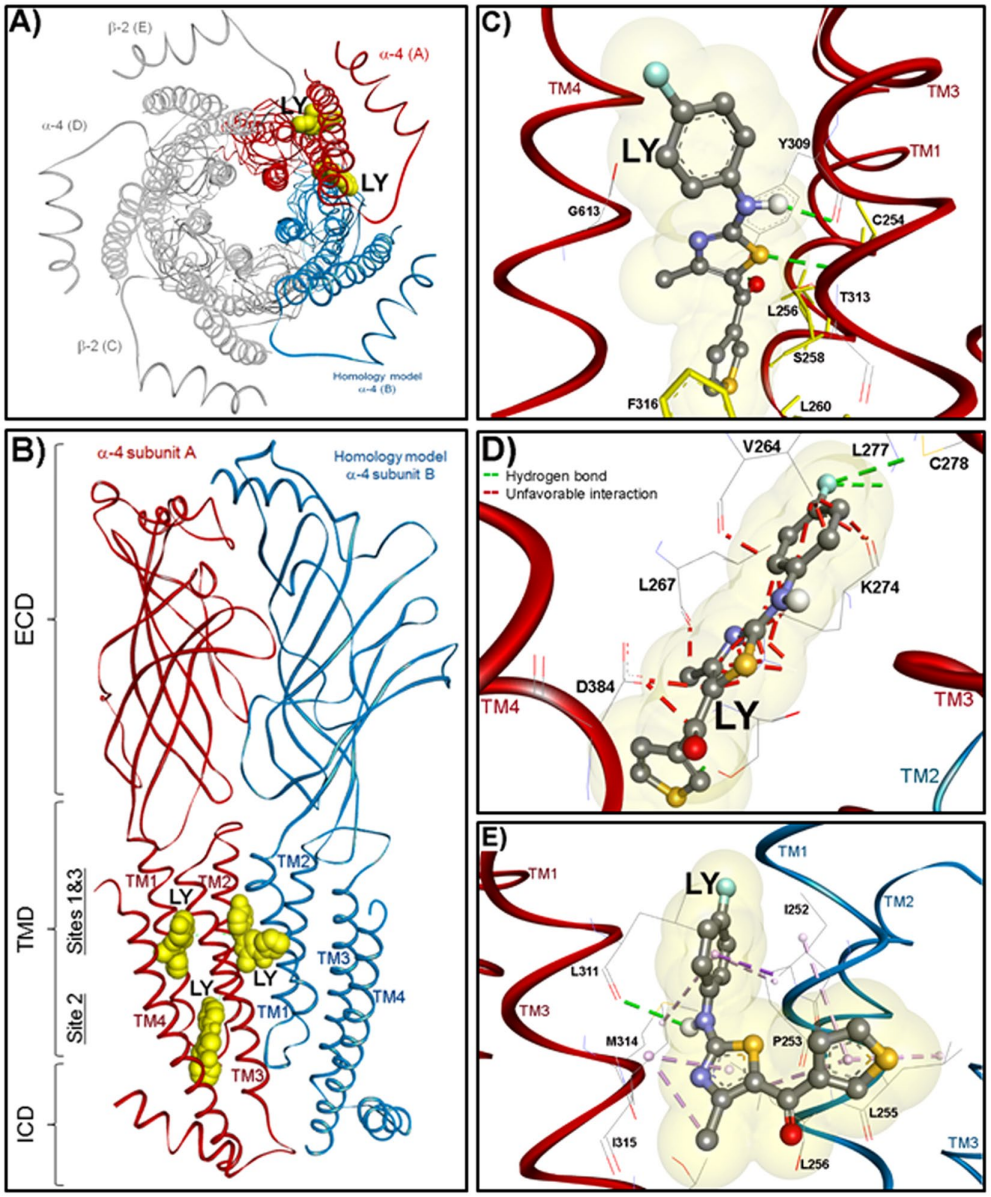
LY2087101 binding sites in the transmembrane domain of (α4)3(β2)2 nAChR. Top (**A**) and Side (**B**–**E**) views showing LY2087101 docked into the (α4)3(β2)2 nAChR at three transmembrane pockets; two within the α4 subunit transmembrane helix bundle (Sites 1 and 2) and one at the α4:α4 transmembrane interface (Site 3) as described under computational docking analyses method section. LY2087101 is shown in yellow space-filling model (**A**,**B**) or in ball and stick format colored by elements (**C**–**E**; Carbone, gray; oxygen, red;). The nAChRs subunits are shown in ribbon with the α4 subunit that provides the (+)face of the α4:α4 interface colored in red and designated as α4 subunit **A**. The α4 subunit that provides the (−)face of the α4:α4 interface (colored in cyan and designated as α4 subunit **B**) does not exist in the crystal structure of (α4)2(β2)3 nAChR (PDB: 5KXI)^6^ and therefore was derived from this crystal structure by homology modeling as described previously^29^. The other nAChR subunits are colored in grey (**B**) or not shown (**A**) for clarity. Details of LY2087101 interactions with amino acids residues within Binding Site 1–3 are shown in **C**–**E**, respectively, with hydrogen bond interactions are shown as green dashed lines, non-bond hydrophobic interactions are shown as violet dashed lines, and unfavorable interactions as red dashed lines.

Mutational analyses of amino acid within the extracellular domain (ECD) of the α4 subunit, particularly those known to contribute to the recognition of NS9283 and CMPI (two PAMs with selectivity to the (α4)3(β2)2 but not (α4)2(β2)3 nAChR)^15,28,29^, did not alter potentiation by LY2087101 and dFBr. This indicates that LY2087101 and dFBr do not share a common binding site with NS9283 or CMPI. However, mutational analyses of amino acid within the transmembrane domain (TMD) of the α4 subunit revealed a set of amino acids that significantly reduced LY2087101 and/or dFBr potentiation when mutated to the corresponding amino acids in the α3 or 5HT3A subunit (Table 1; Supplementary Figure 2). For LY2087101, these critical amino acids include α4Leu256 within the transmembrane helix 1 (TM1), α4Phe316 within the TM3, and α4Gly613 within TM4 and for dFBr critical amino acids include α4Leu256 within TM1, α4Glu308/α4Phe312/α4Phe316 within the TM3, and α4Gly613 within TM4. The location of these amino acids and the effect of mutation at additional positions that project to the space within the α4 subunit transmembrane (TM1-TM4) helix bundle or project to the α4 subunit interfaces lead us to examine three potential binding sites summarized in Fig. 10. Two intrasubunit sites within the α4 subunit TM helix bundle; one toward the extracellular side (Binding Sites 1) and the second toward the intracellular side (Binding Sites 2) and one intersubunit site at the α4:α4 subunit transmembrane interface (Binding Site 3). Computational docking analyses of LY2087101 were performed as described under Methods section and then Gold Score algorithmic function^36^ was implemented to evaluate LY2087101 docked at these three potential binding sites. LY2087101 docking parameters and its predicted interactions with amino acids residues within Binding Site 1–3 are presented in Table 3 and shown in Figure C–E.

**Table 3:** GOLD analyses parameters of flexible docking of LY2087101and dFBr into potential binding site 1–3 in the (α4)3(β2)2 nAChR.

Docking site	PAM	Gold Score Fitness	Gold Score External vdw	Gold Score External HB	Hydrogen bonds	
					Atoms of PAM	Amino acid
Site 1	LY2087101	63.79	49.16	0.55	Thiazole-S *p*-Phenyl-NH Thionyl 3-C=3O	HN of Thr313 O=C of Tyr309 HO of Tyr309
Site 1	dFBr	52.08	39.59	0.17	CH_3_-NH	O=C of V609
Site 2	LY2087101	−25.08	7.57	0.88	Thiophene-S *p*-Phenyl-F *p*-Phenyl-F	HO of S269 HN of C278 HN of L277
Site 2	dFBr	36.47	30.35	0.0	Indole-NH CH_3_-NH CH_3_-NH	O=C of A595O=C of V264O =C of L267
Site 3	LY2087101	55.90	43.19	0.00	*p*-Phenyl-NH	O=C of Leu311
Site 3	dFBr	41.34	33.12	0.00	Indole-NH	SH of C259

### LY2087101 intrasubunit binding site (Binding Site 1)

LY2087101 (Fig. 1) contains a three ring structure, a *p*-fluorophenyl, a 4-methylthiazolyl, and a thienyl moieties. Computational docking of LY2087101 into the potential Binding Site 1 within the α4 subunit helix bundle formed by amino acid residues from TM1-TM4 was performed using the crystal structure of the human (α4)2(β2)3 nAChR (PDB: 5kxi)^6^. The results of docking analyses predicted stable binding of LY2087101 within the upper part (close to the extracellular end) of α4 subunit helix bundle with a Gold Score fitness of 63.79 (Fig. 10C). In the most energetically stable binding mode of LY2087101, the thienyl moiety is positioned in the center of a coordinate formed by α4Ser258 from TM1, α4Thr313 from TM3, and α4Cys610 from TM4 and the rest of LY2087101 molecule extends diagonally (upward and outward) along its long axis between the TM3 and TM4 with the a *p*-fluorophenyl moiety ends between α4Phe312 (TM3) and α4Gly613 (TM4). In this binding mode, LY2087101 is in close proximity to critical amino acid residues that when mutated significantly reduced LY2087101 potentiation of (α4)3(β2)2 nAChR (Figs 5–7, Table 1). The 4-methylthiazolyl moiety, which is the center of the LY2087101 structure, was 2.6, 3.5, and 4.3 Å from α4Thr313 (potentiation ratio ***PR***_*T313C*_ = ~2), α4Gly613 (***PR***_*G613L*_ = ~1), and α4Phe316 (***PR***_*F316L*_ = ~1), respectively. Furthermore, the *p*-fluorophenyl moiety of docked LY2087101 was within 5 Å from α4Glu308 (***PR***_*E308V*_ = ~1.7), and α4Phe312 (***PR***_*F312V*_ = ~2.5) and the thienyl moiety was within 5 Å from α4Thr261 (***PR***_*T261D*_ = ~1.6), α4Ser258 (***PR***_*S258M*_ = ~1.5), and α4Phe606 (***PR***_*F606Y*_ = ~2.3). Within Binding Site 1, LY2087101 is predicted to form three hydrogen bond interactions (shown in green dashed lines in Fig. 10C) with α4Tyr309 and α4Thr313 within TM3. Unlike docking at Binding Site 1, LY2087101 docking deeper in the α4 subunit helix bundle below the level of α4Phe606 and closer to the intracellular end of the membrane resulted in poor Gold Score fitness (−25.08) and was associated with unfavorable clash interactions between docked LY2087101 and amino acid residues α4Val264, α4Leu267, α4Lys274, and α4Asp599 within the TM4 (shown as red dashed lines in Fig. 10D).

### LY2087101 intersubunit binding site (Binding Site 3)

The α4:α4 subunit transmembrane interface is formed by TM3 from one α4 subunit (the (+)face) and TM1 from the adjacent α4 subunit (the (−)face) as well as amino acid residues from TM2 and TM4 of both subunits. Because the x-ray structure of (α4)2(β2)3 nAChR (PDB: 5kxi)^6^ does not have a third α4 that provides the (−)face of the α4:α4 interface, it was necessary to use a homology model of the (α4)3(β2)2 nAChR as described in Wang *et al*.^21^ in order to perform LY2087101 docking at Binding Site 3. In agreement with mutational analyses results (Figs 5–7**)**, computational docking of LY2087101 at the α4:α4 TMD interface (Fig. 10E) predicted stable binding of LY2087101 within the upper part (close to the extracellular end) of α4:α4 TMD interface with a Gold Score fitness of 55.90. In its most energetically stable binding mode, the 4-methylthiazolyl moiety is positioned at the level of and within 2.6 Å from α4Ile315 of the TM3 of the α4(+) face (*designated as α4 subunit A in* Fig. 10) and within 1.1 Å from α4Leu256 of the TM1 of the α4(−) face (*designated as α4 subunit B in* Fig. 10). The 4-methylthiazolyl moiety of LY2087101 also in closed proximity to α4Ile252 (2.7 Å) and α4Pro253 (4.1 Å) within the TM1 of the α4(−) face as well as α4Met314 (5.6 Å), α4Phe312 (6.6 Å), and α4Phe316 (8.2 Å) within the TM3 of the α4(+) face. The thienyl ring end of the LY2087101 structure projects outward closer to the α4(−) face and within 3.0 and 4.0 Å from α4Leu255 and α4Ile252. Whereas, the *p*-fluorophenyl moiety end of LY2087101 extends along the axis of the TM1/TM3 ending at the level of α4Leu311 and surrounded by α4Met314 (3.0 Å) and α4Ile315 (3.2 Å) in the TM3 of α4(+)face and within 3 Å from α4Ile248, α4Ile252, α4Pro253 TM1 of the α4(−) face. Within the binding pocket at the α4:α4 TMD interface, LY2087101 is predicted to form one hydrogen bond with α4Leu311 in the α4(+) face and multiple non-bond hydrophobic interactions with α4Met314, and α4Ile315 in the α4(+) face and α4Ile252, α4Pro253, α4Leu255, and α4Leu256 in the α4(−) face. These non-bond hydrophobic interactions can be described as follow: (i) π-σ hydrophobic interactions between *p*-fluorophenyl moiety and α4Ile252; (ii) π-alkyl hydrophobic interactions between *p*- fluorophenyl moiety and α4Pro253 and α4Met314; (iii) π-alkyl hydrophobic interactions between thienyl ring and α4Ile252, α4Leu255, and α4Leu256; (iv) π-alkyl hydrophobic interaction between thienyl ring and α4I315; and (v) Alkyl hydrophobic interaction between 4-methyl group of the 4-methylthiazolyl moiety and α4Ile315. Furthermore, the presence of leucine at position α4311 within the TM3 of the α4(+) face seems to impose a steric impact on LY2087101 (α4Leu311 < 1 Å from *p*-fluorophenyl ring of LY2087101) but not hindering LY2087101 binding. When α4Leu311 was mutated to valine (a shorter side chain but still aliphatic) the effect of LY2087101 was enhanced 3 folds (***R***_*L311V*_ = ~16, Table 1). There is no published study detailing the structure-activity relationship of LY2087101 and other (2-amino-5-keto)thiazol compounds. However, replacement of the thienyl group with tolyl group (LY1078733) or benzodioxolyl (LY2087133) increased the maximum potentiation at α4β2 nAChR^24^ presumably by enhancing non-bond hydrophobic interactions with the predominantly hydrophobic amino acids in the LY2087101 binding pocket at the α4:α4 subunit TMD interface.

### dFBr recognition sites within the transmembrane domain

**Figure 11:**
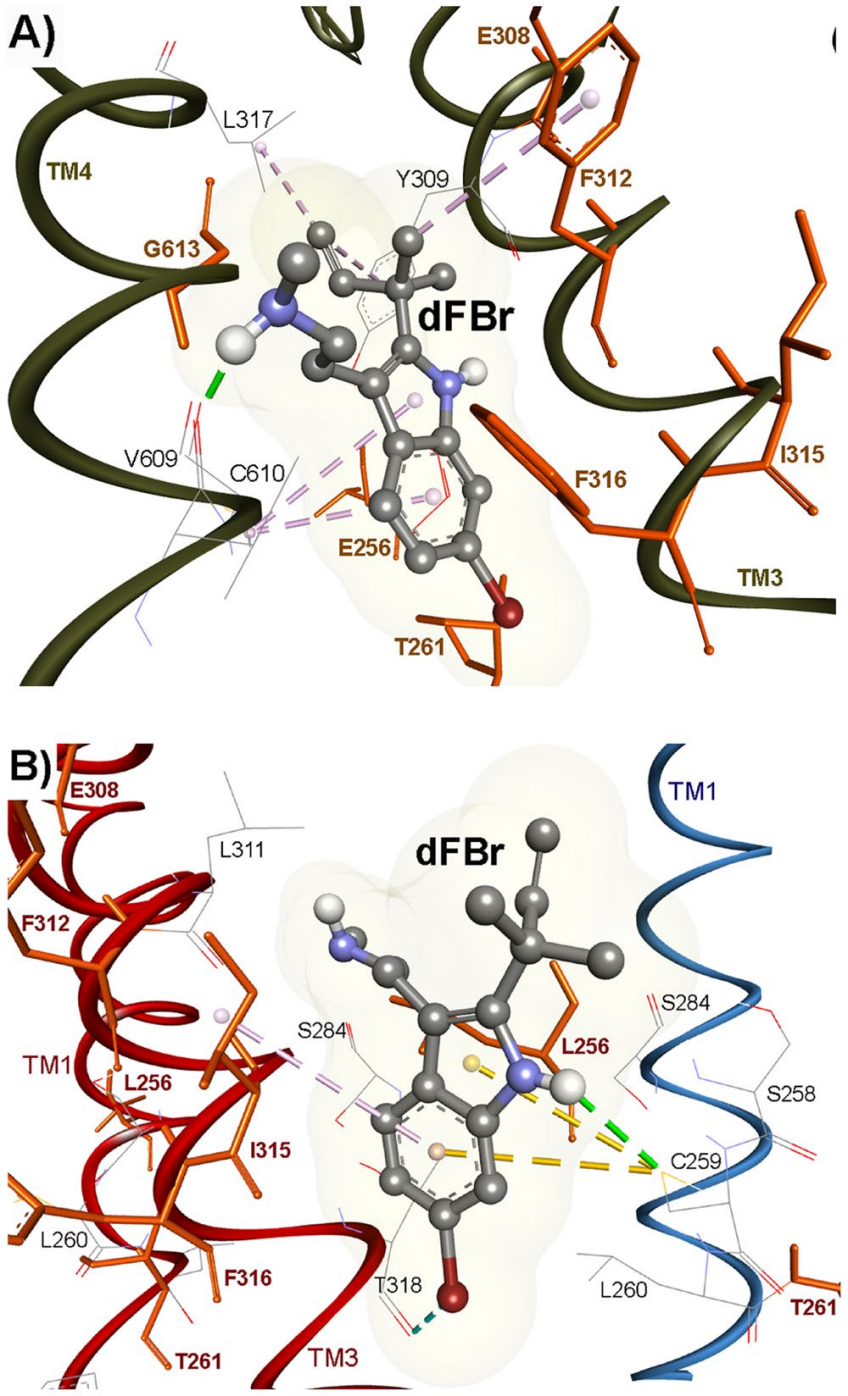
dFBr binding sites in the transmembrane domain of (α4)3(β2)2 nAChR. Side views showing dFBr docked within the α4 subunit transmembrane helix bundle (Binding Site 1, **A**) and at the α4:α4 transmembrane interface (Binding Site 3, **B**). The α4 subunits are shown as ribbon with the subunit that provides the (+)face and (−)face of the α4:α4 interface are colored in red and cyan, respectively. dFBr is shown in ball and stick format colored by element whereas key amino acids side chains are shown in line format. Hydrogen bond interactions and non-bond hydrophobic interactions between dFBr and amino acids residues within Binding Site 1 and 3 are shown as green and violet dashed lines, respectively.

We also studied the effect of amino acid substitution within the LY2087101 intersubunit and intrasubunit binding sites on dFBr potentiation of (α4)3(β2)2 nAChR (Figs 5, 8, and 9; Table 1). We identified 7 amino acid residues within the α4 subunit TMD that significantly reduced dFBr potentiation when mutated to corresponding amino acid in α3 or 5HT3AR subunit: four positions [α4Thr261 (dFBr potentiation ratio, ***PR***_*T261D*_ = ~2.1), α4Phe312 (***PR***_*F312V*_ = ~1.2), α4Phe316 (***PR***_*F316L*_ = ~1.2), and α4Gly613 (***PR***_*G613L*_ = ~0.8)] that project to the α4 subunit helix bundle and contribute to the LY2087101 intrasubunit binding site and three positions [α4Leu256 (***PR***_*L256F*_ = ~1), α4Glu308 (***PR***_*E308V*_ = ~0.8), and α4Ile315 (***PR***_*I315A*_ = ~1.2)] that project to the α4:α4 interface and contribute to the LY2087101 intersubunit binding site. To visualize the binding mode of dFBr we performed dFBr computational docking analyses at potential Binding Sites 1–3 using same experimental strategy and results analyses described above for LY2087101 docking. Similar to LY2087101, dFBr docked within Binding Sites 1 and 3 with favorable highest Gold score fitness of 52.08 and 41.34 **(**Table 3**)**. Within Binding Sites 1 (Fig. 11A), the lowest energy docking solution for dFBr positioned the bromophenyl ring at the level of α4Phe316 (3 Å from 6-Br) and the allyl and methylamine ends of dFBr at the level of α4Gly613 (TM4). In this binding mode, dFBr is also within 5 Å from other amino acid residues identified by mutational analyses (α4Thr261, α4Glu308, α4Phe312) and predicted to form hydrogen bond with α4Val609 and multiple π-alkyl hydrophobic interactions with α4Leu617, α4Tyr309, and α4Phe312. Within Binding Sites 3 (Fig. 11B), the bromoindole ring of dFBr lowest energy docking solution is centered within 4 Å from α4Ile315 (TM3 of the (+)face) and α4Leu256 (TM1 of the (−)face) and the rest of dFBr molecule extends upward placing the N-methylethylamine moiety within 7.7 Å from α4Glu308 (TM3 of the (+)face) and the allyl moiety maintains a close proximity (4.3 Å) to α4Leu256. Docked dFBr is predicted to form a hydrogen bond and two π-Sulfur interactions with SH group of α4Cys259 and π-alkyl hydrophobic interaction with α4Leu311.Within Binding Site 2 (not shown), dFBr docking revealed a Gold Score fitness of 36.47 and hydrogen bond interaction with α4Ile267, α4Val264, and α4Ala595 (Table 3). Nevertheless, the N-methylethylamine moiety of docked dFBr was associated with unfavorable clash interactions with side chains of α4Val264, α4Phe265, α4Leu267, α4Lys274, α4Val327, and α4Ala595 making dFBr docking at Binding Site 2 entirely unfavorable. These results indicate that the two sites we identified for LY2087101 also bind dFBr and emphasize the role α4Thr261 α4Phe316, and α4Gly613 as a common amino acid contact that facilitate recognition of both LY2087101 and dFBr within the α4 subunit helix bundle (Binding Site 1). In similar fashion, α4Leu256 within the TM1 of the α4(−) face and α4Glu308 within the TM3 of the α4(+) face are common recognition amino acids for both LY2087101 and dFBr binding at the α4:α4 TMD interface (Binding Site 3). Still, LY2087101 and dFBr have additional amino acid contacts within these two sites that are unique to each of them. For example, positions α4Phe312 (Binding Site 1) and α4Ile315 (Binding Site 3) mainly influence dFBr potentiation whereas positions α4Ser258 (Binding Site 1) and α4Leu260 (Binding Site 3) mainly influence LY2087101 potentiation. These subtle differences in amino acid recognition of LY2087101 and dFBr may underline the differences in their effects on agonist-mediate responses and nAChR gating kinetics. It is important to mention that the functional contribution of an amino acid side chain can be due to its interaction with dFBr/LY2087101 at the Binding Site 1 (intrasubunit site) and/or Binding Site 3 (intersubunit site). For example α4Phe316 amino acid side chain is accessible from the intersubunit and intrasubunit space and located within 3 and 5 Å from dFBr and LY2087101 docked in Binding Site 1 and within 6 and 8 Å from dFBr and LY2087101 docked Binding Site 3, respectively.

Structural information about the number and location of nAChR PAMs binding sites are emerging and nAChR PAM recognition sites have been identified within the extracellular and transmembrane domains of nAChRs. These include intrasubunit PAM binding sites within the transmembrane domain of a nAChR subunit^27,37^ and intersubunit PAM binding sites at subunits interface within the extracellular and transmembrane domains^28,33,38–40^. The functional consequences of amino acid substitutions we performed on this study on LY2087101 and dFBr potentiation and the location of these amino acids and their predicted interactions with docked LY2087101 identify two sites within the TMD of (α4)3(β2)2 nAChR: one within the upper part of the α4 subunit helix bundle and one at the α4:α4 subunit interface. Our results also establish that LY2087101 binding at these sites is governed by multiple nonbonding interactions with hydrophobic amino acid residues that line these binding sites. Comparing our results of LY2087101 binding to the heteropentameric (α4)3(β2)2 nAChR with that in the homopentameric α7 nAChR reveal equivalent sites within the helix bundle of α4 and α7 subunits with (α4)3(β2)2 and α7 nAChR potentially contain three and five intrasubunit sites, respectively. In addition, the (α4)3(β2)2 nAChR contains an additional intersubunit site that has not been yet identified or does not exist in the α7 nAChR. In the (α4)3(β2)2 nAChR, LY2087101 can bind at one or more of these four possible binding sites per receptor molecule (3 sites within the three α4 subunits and a site at the α4:α4 subunits interface) with occupancy of these sites depends on the concentration of LY2087101 and its relative affinity at these sites. Although additional studies are necessary to determine LY2087101 affinities at these sites and their functional contributions, there was no difference in LY2087101 potency at (α4)3(β2)2 vs. (α4)2(β2)3 nAChR suggesting that LY2087101 bind with similar affinities at intrasubunit and intersubunit sites. In addition, the fact that a point mutation at either site can abolish LY2087101 potentiation suggest that LY2087101 occupancy at either site is not sufficient to enhance ACh-induced channel gating and that LY2087101 fully potentiates (α4)3(β2)2 nAChR by simultaneously occupying intrasubunit and intersubunit binding sites within the transmembrane domain.

## Methods

### Materials

pcDNA1 plasmids with cDNA encoding for human α3 or β4 nAChR subunits and pSP64ployA plasmids with cDNA encoding for human α4 (pSP64ployA) or β2 nAChR subunits were generously provided by Dr. Jon Lindstrom (University of Pennsylvania). Desformylflustrabromine (dFBr; N-(2-[6-bromo-2(1,1-dimethyl-2-propyl)-1H-indol-3-yl]ethyl-N-methylamine), and LY2087101 ([2-[(4-Fluorophenyl)amino]-4-methyl-5-thiazolyl]-3-thienylmethanone) were purchased from (TOCRIS Bioscience, R&D, Minneapolis, MN). Acetylcholine chloride and other chemicals were purchased from Sigma-Aldrich (Milwaukee, WI) unless otherwise indicated in the text. Mutagenic primers for site-directed mutagenesis were designed using PrimerX (http://www.bioinformatics.org/primerx/) and synthesized at Integrated DNA Technologies (Coralville, Iowa).

### Expression of wild-type and mutant nAChRs in *Xenopus* oocytes

For amino acid substitutions within the α4 nAChR subunit, point mutations were introduced into pSP64ployA plasmid with cDNA encoding for human α4 nAChR subunit with mutagenic primer pairs using the Quick Change II Site-Directed Mutagenesis Kit (Agilent Technologies) then confirmed by DNA sequencing (GENEWIZ, LLC, South Plainfield, NJ). cRNA transcripts suitable for oocyte expression were prepared *in vitro* from linearized plasmids [α3 (BamH1), α4 (AseI), β2 (PvuII), β4 (XhoI)] using mMESSAGE mMACHINE high yield capped RNA transcription kits (Ambion) and purified on NucAway Spin column (Invitrogen). cRNA concentration was determined by spectroscopy (Concentration (ug/ul) = Abs260 * 40), aliquoted and stored at −80 °C until used.

Ovarian lobules were surgically harvested from oocytes-positive female *Xenopus laevis* (NASCO, Fort Atkinson, WI) according to an animal use protocol approved by the Texas A&M Health Sciences Center Institutional Animals Care and Use Committee. The Texas A&M Health Sciences Center Research Facility is registered as an animal research facility with the United States Department of Agriculture and is fully accredited by the American Association for Accreditation of Laboratory Animal Care (AAALAC). For oocytes defolliculation, ovarian lobes were treated with 3 mg/ml collagenase type 2 (Worthington Biomedical, Lakewood, NJ) in Ca + 2-free buffer (85 mM NaCl, 2.5 mM KCl, 1 mM MgCl2, 5 mM HEPES, pH 7.6). Collagenase treatment was allowed to proceed for 3 hours at room temperature with gentle shaking then oocytes were washed several times with buffer to remove collagenase then with ND96-gentamicine buffer. Healthy Stage V and VI oocytes were visually selected and maintained at 18 °C in modified ND96-gentamicine buffer (96 mM NaCl, 2 mM KCl, 1.8 mM CaCl2, 1 mM MgCl2, 5 mM HEPES, 50 µg/ml gentamicin, pH 7.6). Oocytes were injected with 50–100 ng of the desired nAChR subunits cRNA mix at ratios of 8α:1β or 1α:8β to express nAChRs with subunit stoichiometries of 3α:2β (low agonist sensitivity) or 2α:3β (high agonist sensitivity), respectively.

### Measurement of ACh responses of WT or mutant nAChRs

Two-electrode voltage clamp recording of ACh-induced responses of *Xenopus* oocytes were performed 24–72 h following injection of nAChR subunit cRNA mix to ensure adequate nAChRs expression. *Xenopus* oocytes were placed in a custom-made recording chamber that is connected to eight channels automated perfusion system (Warner Instruments) and voltage-clamped at −50 mV using Oocyte Clamp OC-725B (Warner Instruments). Oocytes were continuously perfused with recording buffer (100 mM NaCl, 2 mM KCl, 1 mM CaCl2, 0.8 mM MgCl2, 1 mM EGTA, 10 mM Hepes, pH 7.5) except during periods of drug applications. Each recording included several drug applications (10 seconds of ACh with or without LY2087101 or dFBr) separated by 2 min buffer wash intervals and oocytes were washed with buffer for 3–5 min between runs. Currents were digitized using Digidata 1550 A (Axon Instruments) and peak currents were quantified using pCLAMP 10 (Axon Instruments) then normalized and analyzed using Excels 2010 (Microsoft corporation) and SigmaPlot 11.0 (Systat Software).

For assessing the effect of amino acid substitutions on LY2087101 and dFBr potentiation of (α4)3(β2)2 nAChR, a potentiation ratio ***PR*** (peak current amplitude elicited by 10 µM ACh in the presence of 1 µM LY2087101 and dFBr relative to the peak current amplitude elicited by 10 µM ACh alone within same recording run) was determined for each substitution. For calculating the concentration-dependent effect of LY2087101 and dFBr, peak ACh currents in the presence of increasing concentrations of LY2087101 or dFBr were normalized to current elicited by ACh alone within the same recording run. For ACh dose-response curves in absence or presence of 1 µM LY2087101 or dFBr, ACh currents were normalized to current elicited by 1000 μM ACh within the same recording run. Replicas (1–3) from the same oocyte were combined and data from N oocytes were combined (Average ± SEM), were plotted and fit to a 3 parameter Hill equation:

1 $${I}_{x}={I}_{0}+{I}{\rm{\max }}/(1+{(E{C}_{50}/{\rm{X}})}^{{\rm{h}}})$$

where *I*_*x*_ is the normalized ACh current in the presence of LY2087101 or dFBr at concentration x, *Imax* is the maximum potentiation of current; h is the Hill coefficient; and *EC*_50_ is the LY2087101 or dFBr concentration producing 50% of maximal potentiation. *I*_*0*_ = 100 was used to fit LY2087101 and dFBr dose-dependent potentiation of ACh responses, whereas *I*_*0*_ = 0 was used to fit ACh dose-response in the absence and presence of LY2087101 or dFBr.

SigmaPlot 11 (Systat Software Inc.) was used to perform statistical analyses (one-way analysis of variance with the Holm-Sidak Test) to determine the probability (*P*) that calculated ***PR*** differs from no potentiation (***PR*** = 1) and the probability (*P*) that ACh *Imax* in the presence of LY2087101 or dFBr differs from no potentiation (ACh alone, *Imax* = 100) or differ from ACh *Imax* in the presence of LY2087101 or dFBr for WT (α4)3(β2)2 nAChR.

### Computational Docking Analyses

#### GOLD docking parameters and Docking Protocol

GOLD (Genetic Optimization for Ligand Docking) software package, version 5.2.2 (Cambridge Crystallographic Data Centre, Cambridge, U.K.)^36^ was used for the docking study. Discovery Studio 4.1 visualizer was used to further prepare the receptors for docking. The region of interest used for GOLD docking was defined as all the protein residues within the 10 Å of the reference ligands that accompanied the downloaded protein complexes. Default values of speed settings and all other parameters were used for both pose selection and enrichment studies. The input structure was the mol2 file with ligand extracted. The water molecules were deleted. The fitness function was set to the GOLD Score fitness function (Chem Score disabled) with default input and annealing parameters. The Gold Score was opted to select the best docked conformations of the inhibitors in the active site. The best docking poses were selected based on the gold fitness score and the critical interactions reported in the literatures. We used 10 genetic algorithm (GA) docking runs with internal energy offset. For pose reproduction analysis, the radius of the binding pocket was set as the maximal atomic distance from the geometrical center of the ligand plus 3 Å. The top ranked docking pose was retained for the 3D cumulative success rate analysis. Rescoring was conducted with the GOLD rescore option, in which poses would be optimized by the program. The Genetic Algorithm default settings were accepted as population size 100, selection pressure 1.1, number of operations 100,000, number of islands 5, niche size 2, migrate 10, mutate 95, and crossover 95. All other parameters accepted the default settings.

#### Preparing (α4)3(β2)2 nAChR homology model for GOLD docking

The crystal structure of human (α4)2(β2)3 nAChR (PDB code: 5KXI)^6^ was used for docking within the α4 subunit helix bundle. Because there is no published crystal structure of (α4)3(β2)2 nAChR, a homology model of (α4)3(β2)2 nAChR was constructed to use as template for docking at the α4:α4 subunit interface which exist in the (α4)3(β2)2 but not the (α4)2(β2)3 nAChR. The (α4)3(β2)2 nAChR homology model was constructed from the human (α4)2(β2)3 nAChR crystal structure (PDB# 5KXI) as previously described^29^ using the “Superimpose Proteins” tool of the Discovery Studio 2017 molecular modeling package from Accelrys. Briefly, a copy of the crystal structure of α4 subunit was superimposed onto and replaced the third β2 subunit in the crystal structure of (α4)2(β2)3 nAChR by minimizing the distances between pairs the α-carbons of β2W57, β2G116, β2C130, and β2P219 with α4W62, α4G121, α4C135, and α4P227, respectively. Then the generated homology model of (α4)3(β2)2 nAChR was energetically minimized using the conjugate gradient algorithm with restraints to all protein atoms. Additional 1000 steps was then used to minimize the energy of the (α4)3(β2)2 model with no restraints. The energy minimized (α4)3(β2)2 model was used for binding site mapping and small molecule docking studies. For each docking target, crucial amino acids of the three proposed binding sites and flexible residues were identified depending on their proximity to the LY2087101 or dFBr molecule manually placed at the assigned site before running the docking calculation. Using the Accelyrs Discovery Studio visualizer v4.1 client software, all hydrogen atoms were added to the receptor atoms, and the receptor was saved in MOL2 format for docking with Gold. The binding site was defined by including all residues within the flood fill radius 10 Å of the origin for each site as mentioned below. All of free rotamer Library Operation of the selected flexible residues were set at 0(180) 0 (180).

Based on the results of mutational analyses described in the results section of this report, LY2087101 and dFBr docking were performed at three binding pockets: *1) Binding site 1*, which is located within the upper half (toward the extracellular side) of α4 subunit helix bundle and was assigned at the origin of x: 29.84; y: −25.01; z: −8.47 with L284 as a flexible residue (*numbering of amino acids begins from the translational N-terminus of α4 subunit, subtract 26 amino acids to get numbering based on the recently published structure of (α4)2(β2)3 nAChR; PDB# 5KXI*)^6^. *2) Binding site 2*, which is located within the lower half (toward the intracellular side) of α4 subunit helix bundle and was assigned at the origin of x: 17.43; y: −19.01; z: −5.29 with Val264, Lys274, and Val317 as flexible residues; and, *3) Binding site 3*, which is located at α4:α4 subunit transmembrane interface and was assigned at the origin of x: 27.91; y: −20.81; z: −18.19 with Leu256, Leu311, and Thr318 as flexible residues.

#### Preparing a ligand file for GOLD Docking

The 3D structures of LY2087101 and dFBr were constructed using Chem3D Ultra 15.1 software [Cambridge Soft corporation, PerkinElmer, USA (2015)] to obtain standard 3D structures (PDB format), then energetically minimized by using MOPAC with 100 iterations and minimum RMS gradient of 0.10., and saved as SYBYL (MOL2) format for docking using GOLD5.2.2. program.

#### Analyzing the docking results by Accelrys DS

Gold Score algorithmic function was implemented to evaluate LY2087101 and dFBr docked at these three potential binding sites which allow superior docking results than the Chemscore as a scoring function^36^. Gold program outputs a detailed record to the result file of Gold configuration file and Gold result file has the extension “.sd”. The similarity of docked structures is measured by computing the root-mean-square-deviation, RMSD, between the coordinates of the atoms. The docking output results including the output Gold Score fitness, external vdw, and external Hydrogen bond were reported. The top ranked pose with highest Gold Score fitness was analyzed using Accelrys Discovery studio visualized 4.1 was used to reveal the hydrogen bond interaction and binding mode within the binding domain.

## Electronic supplementary material

Supplemenraty Figures
