# Supplementary material for: LY2087101 and dFBr share transmembrane binding sites in the (α4)3(β2)2 Nicotinic Acetylcholine Receptor
Source: Sci Rep. 2018 Jan 19;8:1249. doi: 10.1038/s41598-018-19790-4 (PMC5775429; doi:10.1038/s41598-018-19790-4)
Supplement: Supplementary file 1 — Supplemenraty Figures [file 41598_2018_19790_MOESM1_ESM.pdf]

LY2087101 and dFBr share transmembrane binding sites in the (α4)3(β2)2 Nicotinic Acetylcholine Receptor

Farah Deba, Hamed I. Ali, Abisola Tairu, Kara Ramos, Jihad H. Ali, Ayman K. Hamouda*

Department of Pharmaceutical Sciences, Rangel College of Pharmacy, Texas A&M HSC, Kingsville, TX 78363.

Supplementary Figure 1

		**TM1**
α4 nAChR	240	RRLPLFYTINLIIPCLLISCLTVLVFYLP
α3 nAChR	238	RRLPLFYTINLIIPCLLISFLTVLVFYLP
α7 nAChR	227	RRRTLYYGLNLLIPCVLISALALLVFLLP
$5HT_{3A}$	210	RRRPLFYVVSLLLPSIFLMVMDIVGFYLP
		**TM2**
α4 nAChR	289	SECGEKITLCISVLLSLTVFLLLITEIIP
α3 nAChR	267	SDCGEKVTLCISVLLSLTVFLLVITETIP
α7 nAChR	256	ADSGEKISLGITVLLSLTVFMLLVAEIMP
$5HT_{3A}$	239	PNSGERVSFKITLLLGYSVFLIIVSDTLP
		**TM3**
α4 nAChR	298	STSLVIPLIGEYLLFTMIFVTLSIVITVF
α3 nAChR	396	STSLVIPLIGEYLLFTMIFVTLSIVITVF
α7 nAChR	287	ATSDSVPLIAQYFASTMIINGLSVVVTVI
$5HT_{3A}$	270	ATAIGTPLIGVYFVVCMALLVISLAETIF
		**TM4**
α4 nAChR	591	WKYVAMVIDRIFLWMFIIVCLLGTVGLFL
α3 nAChR	468	WKYVAMVIDRLFLWVFTLVCILGTAGLFL
α7 nAChR	461	WKFAACVVDRLCLMAFSVFTIICTIGILM
$5HT_{3A}$	450	WKRVGSVLDKLLFHIYLLAVLAYSITLVM

Amino acid sequence alignment of the transmembrane domains of α3, α4, and α7 nAChR subunits and the 5HT3-A subunit. Amino acids are represented by a single letter code and the span of the transmembrane helices (TM1-TM4) are indicated by solid bars above the sequence.

Supplementary Figure 2

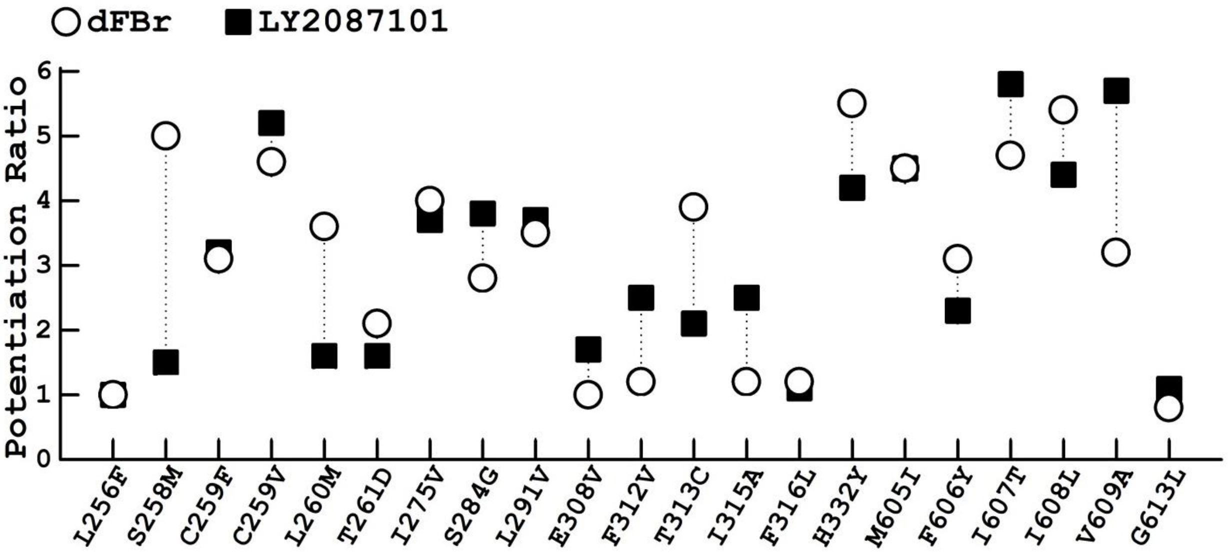

LY2087101 and dFBr potentiation ratios (*PR*) for (α4)3(β2)2 nAChRs containing amino acid substitution within the transmembrane domain of the α4 subunit. LY2087101 and dFBr PRs are calculated as a ration of peak current amplitude in response to application of 10 µM ACh+1 µM LY2087101 or 1 µM dFBr, respectively, relative to peak current amplitude elicited by 10 µM ACh alone. LY2087101 and dFBr potentiation ratios (PR) for wild-type (α4)3(β2)2 nAChRs were 4.1±0.3 and 4.4±0.2, respectively.

Supplementary Figure 3

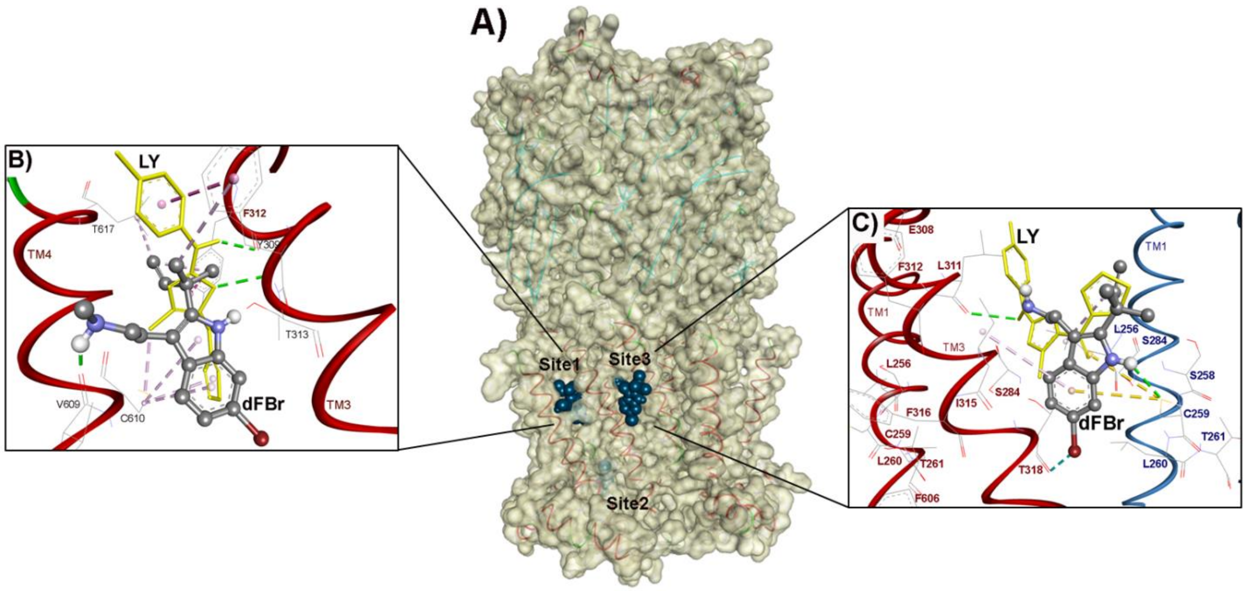

Comparison of dFBr and LY2087101 mode of binding in the (α4)3(β2)2 nAChR. ***A,*** space-filling side view of (α4)3(β2)2 nAChR showing the overall locations of dFBr binding sites (blue space-filling) within the transmembrane domain. ***B and C,*** close up side view showing secondary structure of α4 subunits as ribbon and overly of the lowest energy docking solutions for LY2087101 and dFBr with Binding Site 1 (***B***) and 2 (***C***). LY2087101 is shown in yellow sticks representation and dFBr is shown in ball and stick format and colored by elements. Side chains of key amino acids are shown in line format. Hydrogen bond interactions and non-bond hydrophobic interactions are shown as green and violet dashed lines, respectively.
